# Exogenous application of selenium on sunflower (*Helianthus annuus* L.) to enhance drought stress tolerance by morpho-physiological and biochemical adaptations

**DOI:** 10.3389/fpls.2024.1427420

**Published:** 2024-07-18

**Authors:** Muaz Ameen, Muhammad Anjum Zia, Hussam F. Najeeb Alawadi, Maria Naqve, Athar Mahmood, Ahamad Naeem Shahzad, Bilal Ahmad Khan, Bushra Ahmed Alhammad, Maha Aljabri, Mahmoud F. Seleiman

**Affiliations:** ^1^ Department of Botany, University of Agriculture Faisalabad, Faisalabad, Pakistan; ^2^ Department of Biochemistry, University of Agriculture Faisalabad, Faisalabad, Pakistan; ^3^ College of Agriculture, Al-Qadisiyah University, Diwaniyah, Iraq; ^4^ Department of Agronomy, University of Agriculture Faisalabad, Faisalabad, Pakistan; ^5^ Department of Agronomy, Bahauddin Zakarriya University, Multan, Pakistan; ^6^ Department of Agronomy, College of Agriculture, University of Sargodha, Sargodha, Pakistan; ^7^ Department of Soil and Crop Sciences, Texas A&M University, College Station, TX, United States; ^8^ Biology Department, College of Science and Humanity Studies, Prince Sattam Bin Abdulaziz University, Al Kharj, Riyadh, Saudi Arabia; ^9^ Department of Biology, Faculty of Science, Umm Al-Qura University, Makkah, Saudi Arabia; ^10^ Plant Production Department, College of Food and Agriculture Sciences, King Saud University, Riyadh, Saudi Arabia; ^11^ Department of Crop Sciences, Faculty of Agriculture, Menoufia University, Shibin El Kom, Egypt

**Keywords:** 0ppm Selenium (Se0), 30ppm Selenium (Se1), 60ppm Selenium (Se2), 90ppm Selenium (Se3), Anthocyanin (Ant), ascorbic acid (AsA), calcium ions (Ca2+), carotenoids (Car)

## Abstract

Drought stress poses a significant obstacle to agricultural productivity, particularly in the case of oilseed crops such as sunflower (*Helianthus annuus* L.). Selenium (Se) is a fundamental micronutrient that has been recognized for its ability to enhance plant resilience in the face of various environmental stresses. The FH-770 sunflower variety was cultivated in pots subjected to three stress levels (100% FC, 75% FC, and 50% FC) and four Se application rates (0 ppm, 30 ppm, 60 ppm, and 90 ppm). This research aimed to investigate the effect of exogenously applied Se on morpho-physiological and biochemical attributes of sunflower to improve the drought tolerance. Foliar Se application significantly lowered H_2_O_2_ (hydrogen peroxide; ROS) (20.89%) accumulation that markedly improved glycine betaine (GB) (74.46%) and total soluble protein (Pro) (68.63%), improved the accumulation of ascorbic acid (AA) (25.51%), total phenolics (TP) (39.34%), flavonoids (Flv) (73.16%), and anthocyanin (Ant) (83.73%), and improved the activity of antioxidant system superoxide dismutase (SOD) (157.63%), peroxidase (POD) (100.20%), and catalase (CAT) (49.87%), which ultimately improved sunflower growth by 36.65% during drought stress. Supplemental Se significantly increased shoot Se content (93.86%) and improved calcium (Ca^2+^), potassium (K^+^), and sodium (Na^+^) ions in roots by 36.16%, 42.68%, and 63.40%, respectively. Selenium supplements at lower concentrations (60 and 90 ppm) promoted the growth, development, and biochemical attributes of sunflowers in controlled and water-deficient circumstances. However, selenium treatment improved photosynthetic efficiency, plant growth, enzymatic activities, osmoregulation, biochemical characteristics, and nutrient balance. The mechanisms and molecular processes through which Se induces these modifications need further investigation to be properly identified.

## Graphical Abstract

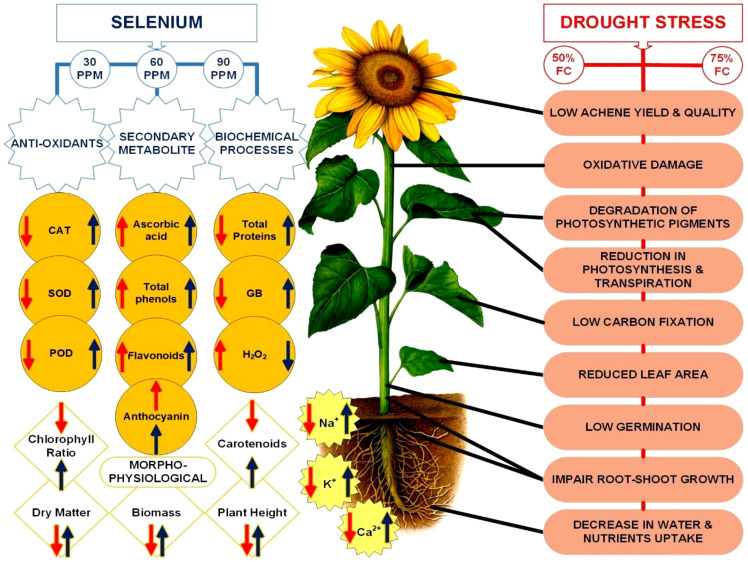

## 1 Introduction

Sunflower (*Helianthus annuus* L.) is a member of the Asteraceae plant family and is grown on 24.77 million hectares worldwide. North America has a long history of cultivating sunflowers, which are now grown globally as an essential oilseed crop. Sunflowers are cultivated in subtropical and tropical countries, where irrigation is traditionally necessary due to drought and extreme temperatures (Arshad et al., 2020). It has 25%–48% oil and 20%–27% protein. It is very adaptable to temperature, moisture, soil composition, and agricultural practices. In Pakistan, area and production are insufficient but crops are commercially important because of their cooking oil yield (Rolnik and Olas, 2021). Nevertheless, drought and insufficient rainfall throughout the growing season substantially lower seed output. According to the USDA Agriculture Weather Facility, drought reduced Spanish sunflower yield by 41%.

Global food security and productivity is severely hampered by climate change and the accelerated growth of the human population (Ameen et al., 2023a). The Intergovernmental Panel on Climate Change (IPCC) Special Report on Climate Change and Land highlights that “Sunflower, a major oilseed crop, is particularly vulnerable to drought conditions, which are expected to increase in frequency and severity under climate change scenarios” (IPCC, 2022a). Crop productivity is occasionally severely affected by a growing number of biogenic and abiogenic challenges, which are imposed on by global warming and other climatic irregularities. Drought stress is caused by a combination of low soil and atmospheric humidity, along with above-average ambient air temperature. According to Mukhtiar et al. (2023), there is an imbalance between the evapotranspiration flux and the soil’s water absorption capacity. The report by the IPCC (2012) provides a framework for managing risks associated with severe events and disasters in order to progress climate change adaptation. The framework highlights the risk that arises from the combination of weather and climate events, as well as vulnerability and exposure.

Climate change is projected to negatively impact crop yields in many regions, with a more significant impact on low-latitude regions (Ameen et al., 2023b). Changes in precipitation patterns due to climate change can lead to water stress, which adversely affects the productivity of oil crops. The IPCC AR5 states, “Drought stress, exacerbated by higher temperatures, can lead to substantial yield reductions in oil crops” (IPCC, 2014). For agricultural productivity and food security, the most serious consequences of climate change are heat and drought. The unfavorable circumstances are negatively impacting plant growth and agricultural productivity of essential crops, leading to the development of regions prone to drought (IPCC, 2022b). Heat and drought processes may be further confounded by a variety of environmental conditions, including both biotic and abiotic components. These problems are undoubtedly significant barriers to agricultural productivity because of oxidative, osmotic, and heat stress brought on by soil salinity and water scarcity (Ahmad et al., 2024; Ameen et al., 2024). Furthermore, when leaf temperatures rise, it has been observed that the reduced stomatal conductance and transpiration under these conditions may result in heat stress. Deficiency of water and elevated temperatures have a major negative impact on plant growth and performance in tropical and subtropical regions (Dietz et al., 2021). Because of the adverse impacts of climate change and increasing drought conditions, this research was conducted to investigate the role of selenium in mitigating drought stress and enhancing the resilience and yield of sunflower crop.

Several factors can lead to water stress in plants, including extreme dry wind seasons, high temperatures, light intensity, and increased evapotranspiration. Sunflower seeds are the fifth-largest edible oil source worldwide. Despite their unrealized potential, they remain a valuable part of a balanced diet or nutraceutical due to their nutritional and therapeutic properties (Bhatt and Patel, 2020). Sunflower’s nutraceutical qualities come from its vitamins, minerals, and phytochemicals such as tocopherols, phytosterols, and flavonoids (Flv). Sunflower oil is cholesterol-free, oxidation-resistant, and rich in linoleic acid. In arid and semi-arid countries with changing climates, drought-tolerant genotypes and adaptive management practices are essential for maintaining sunflower yield.

Water stress is a major factor affecting plant development. Typically, living plant cells contain 85%–95% water. Plants have three distinct types of water associations. The main type of water is bound water molecules, which are chemically linked to complexes such as salts and organic substances. This water is not easily transferred when required or when the plant is under stress (Zafar et al., 2023). Surface water is the second type that occurs on amphiphilic substances including proteins, lipids, and fats. Surface water contributes to 5%–10% of the water content in plant body tissue, but it is uncommon since it penetrates protoplast structures like plasma membranes (Ivanov et al., 2019). Capillary characteristics, hydrogen bonding, and imbibition enable the binding of surface water molecules. The final kind is water that flows freely across plant cells and makes up almost 50% of the plants’ water content. This water moves by osmotic influences in tissues (Munns et al., 2020).

Available nutrients and water affect plant development and agricultural yield. Limited CO_2_ uptake causes an excess of decrease in the photosynthetic electron chain in plants under water stress. Particularly in the photosynthetic and mitochondrial electron transport chains, this excess reducing power directs light energy toward mechanisms that promote the formation of reactive oxygen species (ROS) (Gull et al., 2023). Water deficit causes oxidative stress in plant cells due to increased electron leakage towards O_2_ during photosynthesis and respiratory activities, resulting to more ROS generation. The O_2_^−^, H_2_O_2_, and OH radicals may specifically target membrane lipids, impair nucleic acids, and disrupt essential biochemical processes, leading to cell death. Non enzymatic and enzymatic antioxidants effectively remove ROS mitigating damage to the cell.

Superoxide dismutase (SOD), an enzymatic antioxidant protective mechanism, is present in numerous cell divisions, catalyzing the disproportion of two O_2_^−^ radicals to H_2_O_2_ and O_2_. Catalases (CAT) and peroxidases (POD) convert hydrogen peroxide (H_2_O_2_) to water (Arikan et al., 2022). Furthermore, normal cell metabolic activities produce ROS by-products. Normal cell metabolism regulates ROS formation and degradation. In severe circumstances, ROS generation overwhelms scavenging mechanisms, causing oxidative stress. ROS can destroy essential biomolecules, impair cellular metabolism, and lead to cell death (Kalyane et al., 2022).

Se is rare in nature; volcanism is its significant source; hence, it is lacking in most soils. It is oxidized like sulfur (SO_4_^2−^, SO_3_^2−^) and is taken up by plants as SeO_4_^2−^ (selenate) and SeO_3_^2−^ (selenite). It is a potent antioxidant and is both a non-enzymatic and an enzyme antioxidant supplement. This nutrient deficiency causes heart attack, angina, and stroke in humans. Selenium deficiency in animals can cause white muscle disease. Animals with this condition may lose hair and muscular mass (Uddin et al., 2022).

From the most recent theories on transport, selenium activates aquaporins, boosting a plant’s absorption of water. Aquaporins are channels that help move water and other biochemical substances across plasma membranes (Sharma et al., 2021). Aquaporins consist of a system of cysteine and methionine complex amino acids. Selenium forms selenocysteine and selenomethionine from these amino acids (Zhang and Chu, 2022). The thiol group’s charge and hydrogen bonds facilitate the movement of water. Selenocysteine is a lately identified amino acid. Selenium substitutes sulfur and make selenocysteine the 21st amino acid (Pedron et al., 2023). This acid is commonly prescribed to reduce breast cancer since it confers resistance to oxidative stress. Selenium releases electrons, then replenishes electrons lost from ROS created by cell stress, reducing their destructive capabilities (Lopez-Cantu et al., 2022).

Since Se has the capability to regulate water levels even under drought conditions, it has been shown that even minute amounts of this element can increase selenium content in lettuce and accelerate the plant’s average growth rate (Hossain et al., 2021). Se is an inexpensive way to improve drought resistance and reduce agricultural losses caused by water-restricting factors. Many agricultural experts have shown interest in selenium in recent decades due to its antioxidant and physiological attributes (Sarkar et al., 2023). Se reduces agricultural losses under various physiological conditions, even though it does not directly impact plant metabolic processes (Kamran et al., 2020). Nevertheless, there has not been much research on the role that Se plays in enhancing drought resistant in plants (Ghorai et al., 2022). By triggering plants’ antioxidant mechanisms during drought stress, Se can manage water balance and improve biomass yield. Even in exceptionally low amounts, Se can enhance plant resistance to drought, promote growth, increase leaf carotenoids and chlorophyll, regulate water availability, accumulate suitable solutes, and activate the antioxidant mechanism (Zahedi et al., 2023).

The main objective of current research was to determine the role of exogenously applied Se on morpho-physiological and biochemical attributes of sunflowers as well as to observe which level of selenium treatment is the best to mitigate the drought stress. Furthermore, we also evaluated the impact of exogenous Se on antioxidants, secondary metabolites, ion uptake, and osmotic regulation.

## 2 Materials and methods

The experiment was undertaken during winter seasons (2022–2023) at the experimental area of the Botanical Garden of University of Agriculture Faisalabad, Community College PARS. The sunflower seeds of the FH-770 variety were obtained from the Department of Oilseeds at the Ayub Agricultural Research Institute (AARI) located in Faisalabad. The experiment was conducted in an open-air environment to simulate natural drought conditions for 18 weeks. The meteorological data and soil physicochemical properties of the experiment site are given in **Tables 1**, **2**, respectively. Soil was collected, air-dried, and sieved to remove debris. The initial moisture content of the soil was calculated by weighing 100 g of fresh soil, which was then oven dried at 105°C for 24 h, and the soil dry weight was measured. Soil moisture content was calculated using the following formula: soil moisture content (%) = (soil fresh weight−soil dry weight/soil fresh weight) ×100.

**Table 1:** Meteorological data of experimental site of Community College PARS, University of Agriculture Faisalabad (2023).

Months	Avg T_max_ (°C)	Avg T_min_ (°C)	Avg RH (%)	ppt (mm/month)	Avg WS (m/s)
February	29.51129	15.71355	47.94161	66.25	1.344839
March	34.65533	19.15667	42.20367	27.48	1.486667
April	37.97567	23.23	39.02733	54.9	1.83
May	40.46	28.543	45.11267	134.77	2.007333
June	36.13433	27.51067	69.88367	235.66	1.910333
July	39.30067	28.57967	56.03167	39.65	1.716667
August	39.19533	26.862	51.43167	72.69	1.721667
September	33.99533	19.531	49.76667	46.42	1.408333
October	27.55133	15.43067	53.95267	17.39	1.102667
November	23.19029	9.616	48.51314	0.63	1.141429

ppt, precipitation; RH, average relative humidity; Avg T_max_, average maximum temperature; Avg T_min_, average minimum temperature; and Avg WS, average wind speed.

**Table 2:** Soil physicochemical traits and soil irrigation treatments of experimental site.

Soil physicochemical traits		
Silt (%)	25.5	(Shirazi and Boersma, 1984)
Sand (%)	59.3	–
Clay (%)	22.6	–
Soil texture	Sandy loam	–
pH	7.4	(Nelson and Sommers, 1982)
EC (dSm^−1^)	1.33	(Nadler and Frenkel, 1980)
FC (%)	10.5	(Khorasaninejad et al., 2011)
N (%)	0.03	(Bremner, 1960)
P (ppm)	7.8	(Chapman and Pratt, 1962)
K (ppm)	125	(Mehlich, 1953)
Soil irrigation treatments		
**Field capacity level**	**Average water volume (mL)**	**Total soil weight (kg)**
100% FC	735	7.7
75% FC	551	7.5
50% FC	367	7.3

EC, electrical conductivity; FC, field capacity; K, potassium; N, nitrogen; and P, phosphorus.

Irrigation treatments were applied to maintain the soil moisture by following the method of Khorasaninejad et al. (2011). Soil moisture was monitored daily using a soil moisture probe (T10 Soil Moisture Meter, XLUX^®^). The pots were regularly weighed, and irrigation was applied whenever the soil moisture content dropped below the designated levels for each treatment. The frequency of irrigation was varied depending on the prevailing weather conditions and the rate of evapotranspiration. The recommended dosage of fertilizer by AARI was applied to meet the nutritional requirements.

**Figure 1:**
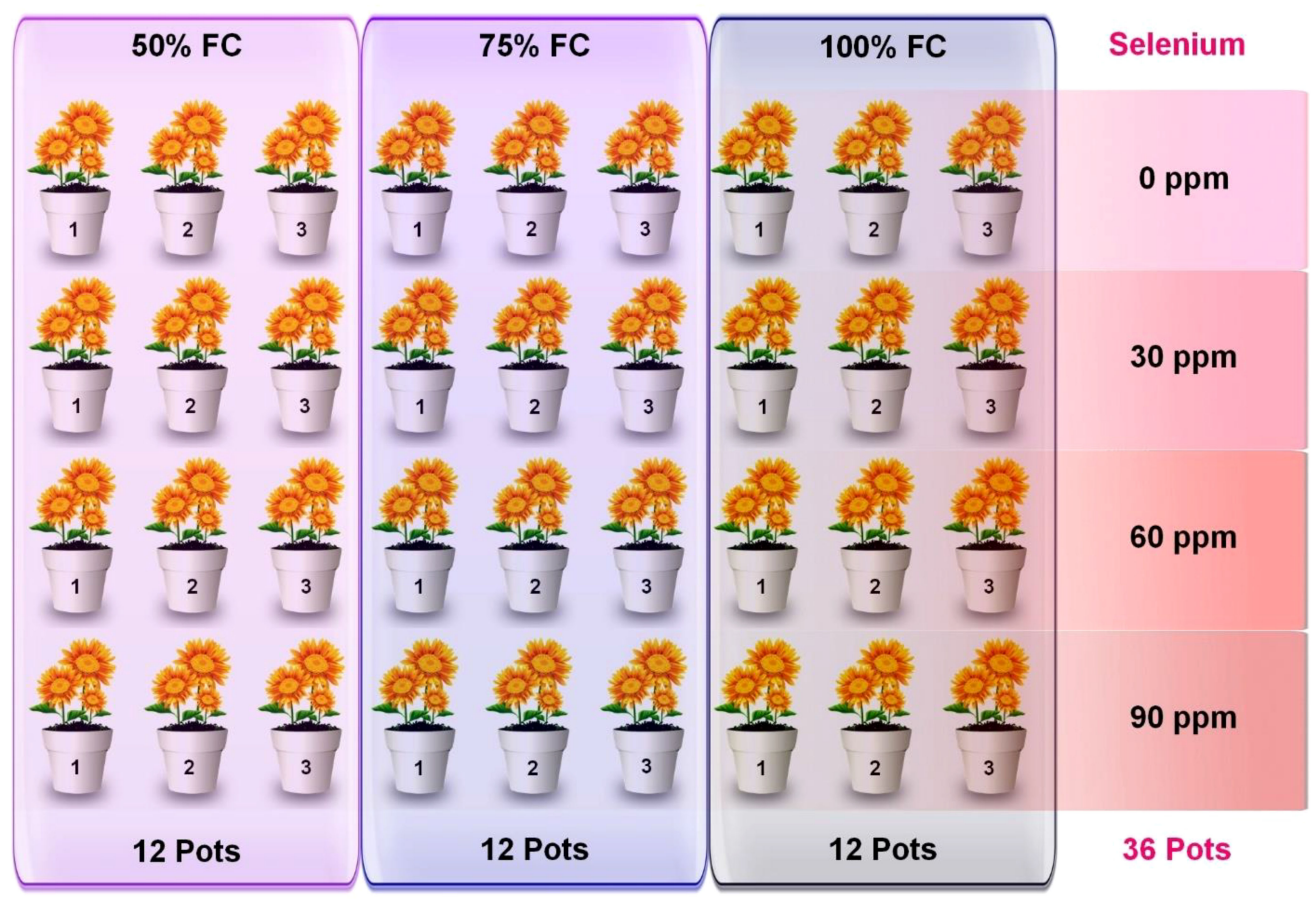
Graphical Illustration of experimental setup.

For this experiment, the plants were sown in standard plastic pots (9.5 cm in diameter × 25.5 cm in height) containing dry soil. A total of 36 pots were distributed in a complete randomized block design with three replicates of each treatment (**Figure 1**) and regularly rotated to reduce the impact of environmental variability. The primary pots (selenium treated) were divided into three irrigation levels, including full irrigation (DS0) with 100% FC, moderate water stress (DS1) with 75% FC, and severe water stress (DS2) with 50% FC. Drought was applied to selective pots after 1 week of the four-leaf stage. Additionally, four rates of selenium application were used, consisting of Se0 (0 ppm), Se1 (30 ppm), Se2 (60 ppm), and Se3 (90 ppm), with Na_2_SeO_4_ (selenate) being the source of selenium. Selenium was applied after 1 week of drought treatment. Data of several morpho-physiological, biochemical, ionic, and enzymatic parameters were analyzed after 3 to 4 weeks of selenium application.

### 2.1 Morphological attributes

The morphological attributes including shoot and root length (SL and RL) (cm), number of leaves (NOL) (plant^−1^), number of branches (NOB) (plant^−1^), shoot and root fresh weight (SFW and RFW) (g), shoot and root dry weight (SDW and RDW) (g), maximum plant height (PH) (cm), total dry matter (TDM) (g), and total plant biomass (PBM) (g) were recorded (Ahmad et al., 2024).

### 2.2 Physiological attributes

The following physiological attributes were calculated.

#### 2.2.1 Root and shoot water content

The water content of the root and shoot (rWC and sWC) was calculated by following the methodology of Barrs and Weatherley (1962). The root and shoot samples were weighed immediately after harvesting to determine their fresh weights (FW). To determine the samples’ dry weights (DW), they were subsequently dried for 48 h at 70°C in an oven. Water content was calculated using the following formula:

Water content = 100 × (FW-DW)/FW

#### 2.2.2 Chlorophyll contents

Chlorophyll content of one plant from each replication was measured by using Davis (1976) and Arnon (1949) methods. One fresh leaf from each replication was collected and 0.5 g of leaf was ground into mortar and pestles. After this, 5 mL of 80% acetone was added to the extract and kept for one night at 10°C temperature. On the next day, the extract was centrifuged at 14,000 rpm value for 5 min. Supernatant absorbance was measured by using a spectrophotometer (InnoTech, Inno-UV2000, China) at three wavelengths 480 nm, 645 nm, and 663 nm for carotenoids, chlorophyll *b*, and chlorophyll *a*, respectively, by using the formula given by Naseem et al. (2023).

### 2.3 Ionic and nutrient analysis

#### 2.3.1 Ions test (Na^+^, Ca^2+^, K^+^)

Wolf (1982) digestion technique was used to measure root mineral ions. In digestion flasks, 0.1 g of dry root from each plant replication was digested in 2.5 mL of concentrated H_2_SO_4_. Four milliliters of 35% H_2_O_2_ was added to the flask and heated at 350°C until colorless. After filtering, this solution was diluted to 50 mL with distilled water. The ions (Na^+^, Ca^2+^, K^+^) in this filtrate were measured using a flame photometer (Jenway, PFP-7, Staffordshire, UK).

#### 2.3.2 Selenium content

Selenium content was determined according to the proposed method of Revanasiddappa and Kiran Kumar (2002). A 5-g plant matter was dissolved by heating with 10 mL of HNO_3_ for 20 min. Then, 0.5 mL of HClO_4_ was added, and the mixture was heated for another 10 min. Water and HCl were used for sterilizing the residues, and it was further boiled for 10 min. The residues were neutralized with NaOH. Then, a 5% EDTA solution was added, and the mixture was diluted to 50 mL. HCL and KI were used to evaluate a 3-mL sample of the solution for selenium. The 600-nm absorbance of liberated iodine was measured using a spectrophotometer (InnoTech, Inno-UV2000, China) after thionine reaction and the concentration of selenium was determined through subtracting the blank absorption.

### 2.4 Secondary metabolites

#### 2.4.1 Total phenolics

To determine total phenol content, Singleton et al (1965) methodology was used to heat 400 mg of dried leaves in 40 mL of ethanol (60%) for 10 min at 60°C and then filtered. Folin-Ciocalteau solution (10 mL) was added to the filtrate (2 mL) and the mixture was left to develop for 5 min. After adding 8.25 mL of 7.5% Na_2_CO_3_, it was incubated for 2 h. Using the regression equation from gallic acid, total phenolics (TP) was calculated after taking the absorption at 765 nm by using a spectrophotometer (Peak Instruments, C7200S, USA).

#### 2.4.2 Ascorbic acid

Trichloroacetic acid (TCA) at a concentration of 6% was used to standardize a leaf sample (0.5 g). According to Mukherjee and Choudhuri (1983), extract was homogenized with 2 mL of 2,4 dinitrophenyl hydrazine and 1 drop of 10% thiourea.

#### 2.4.3 Flavonoid

Flv content was measured by following Zhishen et al (1999) methodology, dipping 100 mg of dried leaves in pure methanol and filtering the extract. This filtrate (1 mL) was incubated for 5 min with distilled water (4 mL), 5% NaNO_2_ (0.3 mL), and 10% AlCl_3_. After incubation, 4% NaOH (2 mL) and distilled water (2.4 mL) were added until they became pink. The usual regression equation was used to figure out rutin after taking absorbance at 510 nm by using a spectrophotometer (Peak Instruments, C7200S, USA).

#### 2.4.4 Anthocyanins

The Flv content was determined by blending fresh leaves in a mixture of HCl, distilled water, and methanol with a 1:20:79 ratio (Mancinelli, 1984). The homogenates were centrifuged at a speed of 13,000 rpm for 20 min at a temperature of 4°C, and the absorbances of the supernatants were measured at 530 and 657 nm on a spectrophotometer (Peak Instruments, C7200S, USA).

### 2.4 Biochemical parameters

#### 2.4.1 Total soluble protein

According to the Bradford (1976) methodology, the total soluble protein (Pro) was determined. Fresh leaf samples (1 g) were homogenized in 4 mL of pH 7.5 sodium phosphate buffer and centrifuged at 4,000 rpm. At 595 nm, a spectrophotometer (Peak Instruments, C7200S, USA) was used to measure the absorbances of the supernatants.

### 2.5 Enzymatic antioxidants

#### 2.5.1 Peroxidase and catalase

According to Chance and Maehly (1955), reaction solutions were made individually for each enzyme. Fresh leaves (0.25 g) were ground with 5 mL of 50 mM potassium phosphate (KP) buffer. The mixture was centrifuged for 15 min at 14,000 rpm to prepare enzyme extract.

For the determination of CAT, the 0.059 M H_2_O_2_ solution was prepared by adding 29 µL of H_2_O_2_ to falcon tubes covered with aluminum foil, raising the volume with distilled water up to 5 mL. Before measuring the absorbance at 240 nm, 100 µL of enzyme extract, 100 µL of H_2_O_2_ (0.059 M), and 1.9 mL of KP buffer (50 mM) were mixed. To determine POD activity, 750 µL of the 50 mM KP buffer was mixed with 100 µL of enzyme extract, H_2_O_2_, and guaiacol, and the absorption of the mixture was recorded at 450 nm by using a spectrophotometer (Peak Instruments, C7200S, USA).

#### 2.5.2 Superoxide dismutase

The method developed by Giannopolitis and Ries (1977) was used to prepare the SOD enzyme samples. The enzyme extract samples were prepared by grinding 0.25 g of fresh leaf and adding 5 mL of 50 mM KP buffer. After that, the sample was centrifuged at 14,000 rpm for 15 min. A blank was prepared with 50 µL of distilled water and 50 µL of KP buffer. Riboflavin was added along with other cuvettes after being exposed to light for 15 min. The SOD enzyme mixture contains 50 µL of enzyme extract, 50 µL of riboflavin, 50 µL of NBT, 250 µL of 200 mM KP buffer, 400 µL of distilled water, 100 µL of Triton, and 100 µL of L-methionine, and the absorbance was measured at 560 nm on a spectrophotometer (Peak Instruments, C7200S, USA).

### 2.6 Organic osmotica and reactive oxygen species

#### 2.6.1 Glycine betaine

The glycine betaine (GB) content was calculated using the methodology of Grieve and Grattan (1983). The homogenate was treated with HCl, KI_3_, and 1,2-dichloroethane in various phases, and the optical density was measured at 365 nm on a spectrophotometer (Peak Instruments, C7200S, USA).

#### 2.6.2 Hydrogen peroxide

Specifically, the methods described by Alexieva et al. (2001) were used to determine H_2_0_2_ content. A 0.5-g sample of fresh leaves was taken and mixed with 5 mL of TCA, 2 mL of KI, and 100 mM KP buffer, and the absorbance at 390 nm was measured on a spectrophotometer (Peak Instruments, C7200S, USA).

### 2.7 Statistical analysis

Analysis of variance of data for all the parameters was calculated under a completely randomized design (CRD) with three replications and mean values by using CoStat-CoHort Software and Statistix 10 Analytical Software following Snedecor (1956) methodology. Graphical illustrations and diagrams were created using Sigma Plot and Microsoft Visio, while R was utilized for generating heatmaps, conducting principal component analysis (PCA), and analyzing correlations.

## 3 Results

### 3.1 Morphological characters

**Figure 2:**
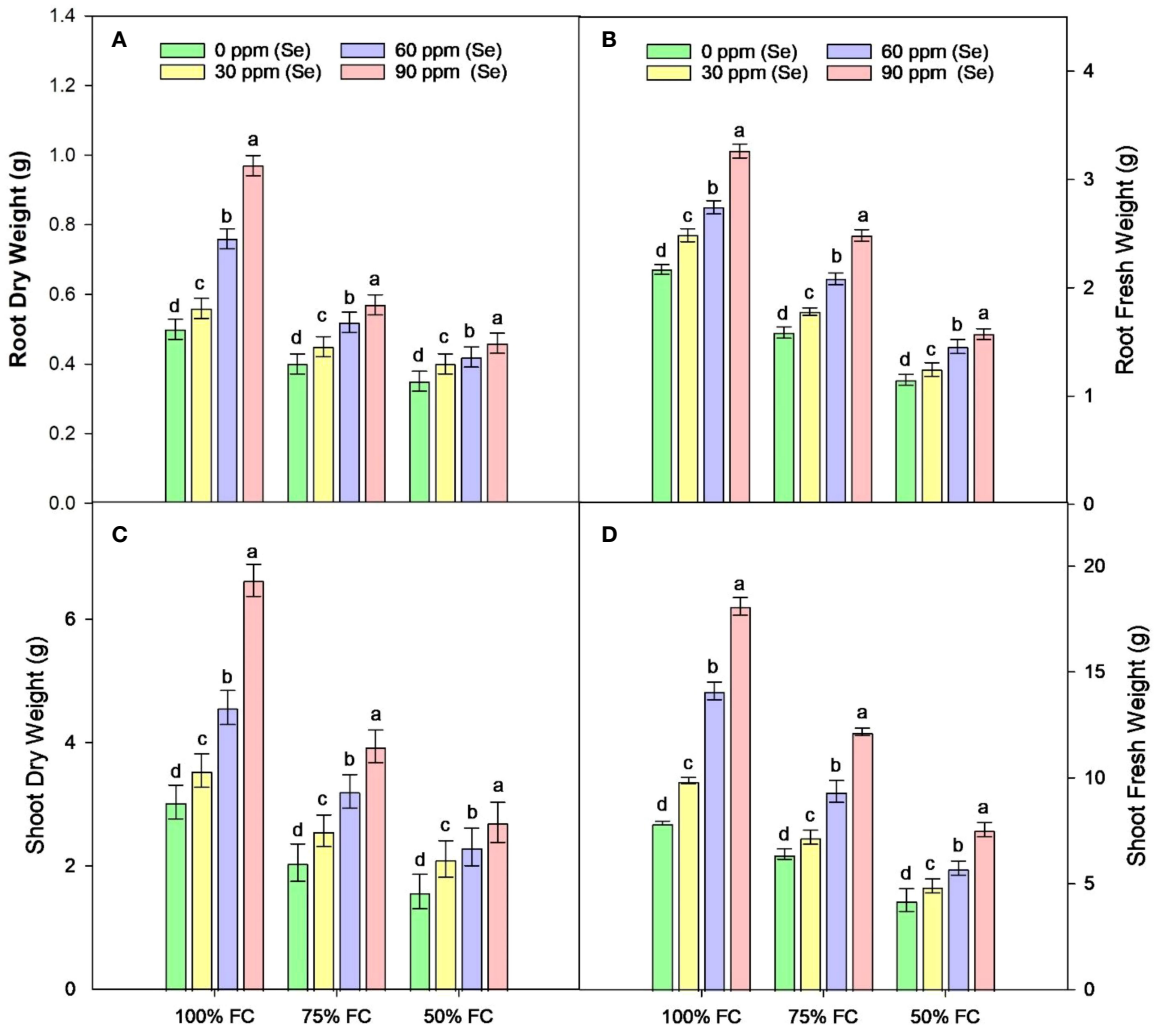
Effect of Se foliar applications on the root dry weight **(A)**, root fresh weight **(B)**, shoot dry weight **(C)**, and shoot fresh weight **(D)** of sunflower during drought stress (*n*=3, mean ± SE). The letters (A, B, C, D) represents the respective graph of the different parameters.

**Figure 3:**
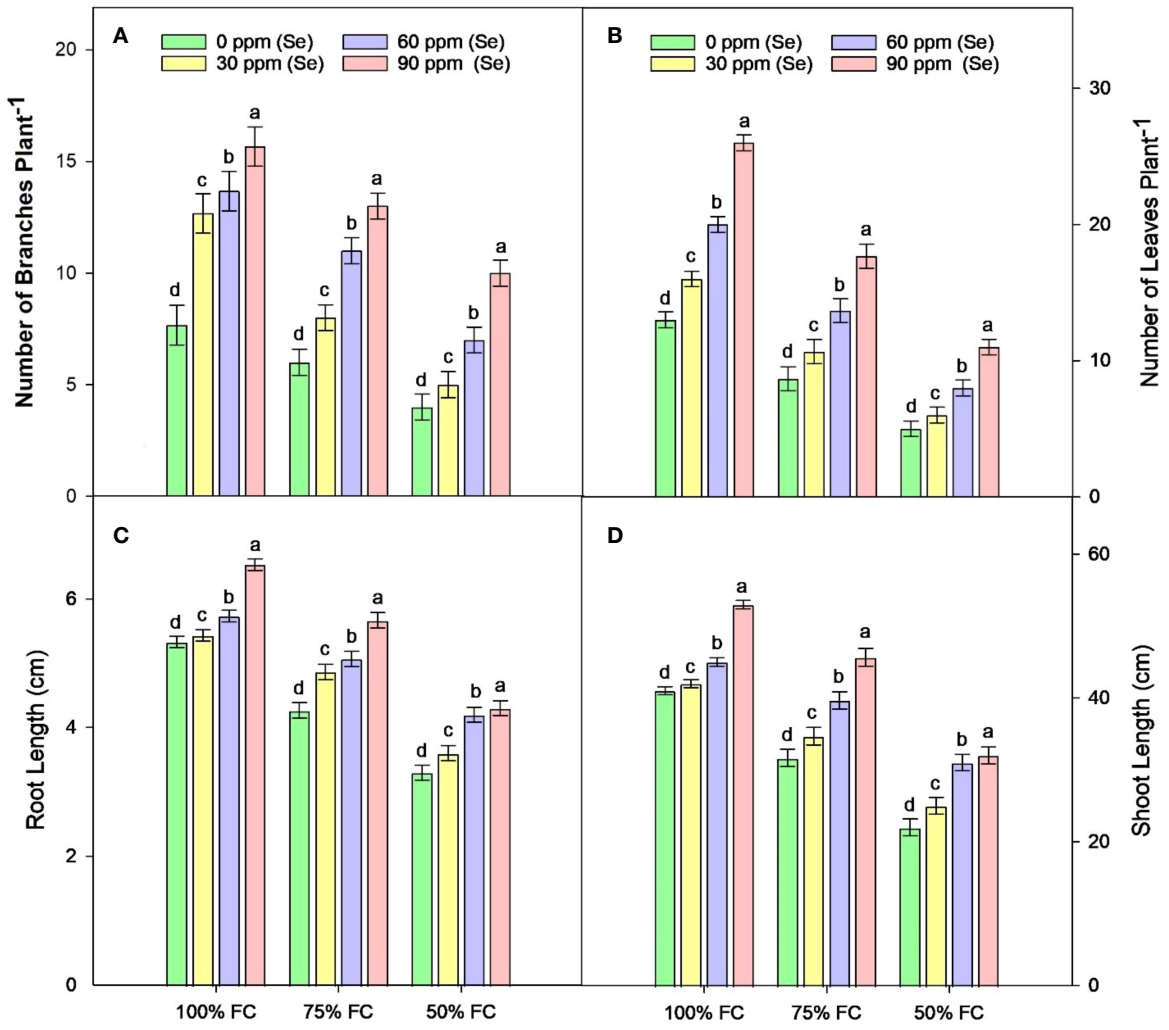
Effect of Se foliar applications on the number of branches **(A)**, number of leaves **(B)**, root length **(C)**, and shoot length **(D)** of sunflower during drought stress (*n*=3, mean ± SE). The letters (A, B, C, D) represents the respective graph of the different parameters.

**Figure 4:**
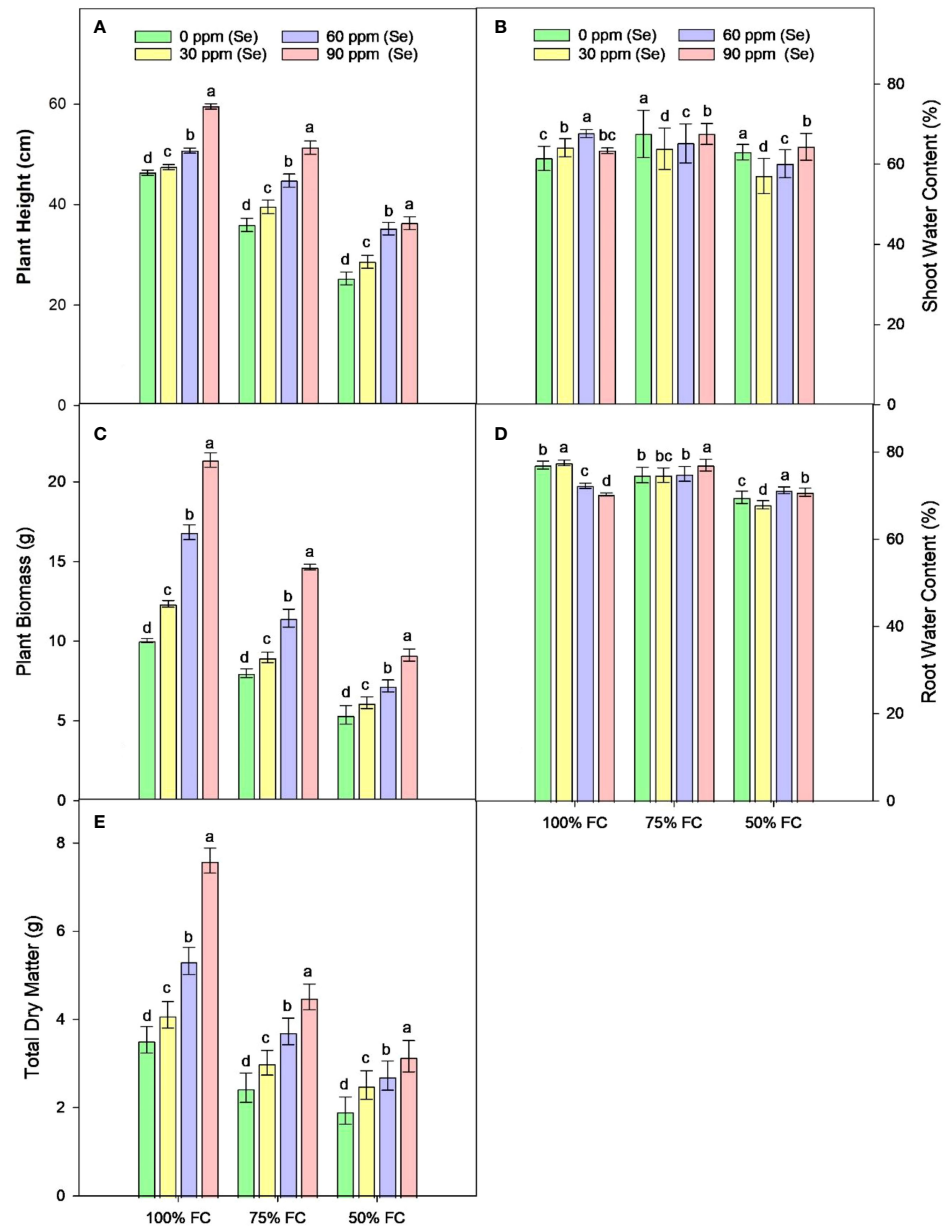
Effect of Se foliar applications on the plant height **(A)**, shoot water content **(B)**, plant biomass **(C)**, root water content **(D)**, and total dry matter **(E)** of sunflower during drought stress (*n*=3, mean ± SE). The letters (A, B, C, D) represents the respective graph of the different parameters.

Sunflower plants with 75% FC and 50% FC exhibited significantly (*p* ≈ 0.0002) decreased SDW (33.83% and 51.07%, respectively), SFW (29.70% and 55.08%, respectively), PH (15.92% and 38.51%, respectively), NOB (23.45% and 47.60%, respectively), and NOL (32.32% and 60%, respectively), compared to 100% FC. For Se applications, all levels of Se (30, 60, and 90 ppm) resulted in increased NOL (22.50% to 104.61%), SDW (23.19% to 98.95%), and SFW (18.71% to 104.32%) compared to the control. The 90-ppm Se was highly effective compared to the 60-ppm Se, increasing the PBM by 92.83%, PDM by 92.76%, and plant height by 36.78%. For combined treatments under 75% FC, the best treatment was 75% FC × Se90. This combined treatment increased RDW, RFW, SFW, and SDW by 42.24%, 56.43%, 90.02%, and 92.00%, respectively (75% FC × Se0) (**Table 3**; **Figures 2**–**4**).

**Table 3:** The statistical analysis (ANOVA) showing mean square values of Se applications on morpho-physiological attributes of sunflower during drought stress.

Source	df	RL	SL	PH	NOB	NOL	RFW	SFW	SDW	RDW	PBM	TDM	sWC	rWC	CHL*a*	CHL*b*	CAR	CHLR	TCHL
Main effects																			
**DS**	2	11.03 ***	954.7 ***	1,170.9 ***	105.0 ***	380.5 ***	5.1 ***	142.3 ***	15.9 ***	0.27 ***	201.5 ***	20.4 ***	73.5 ns	100.3 ***	3.1 ***	1.1 ***	1.7 ***	6.5 ***	4.9 ***
**Se**	3	2.4 ***	254 ***	305.4 ***	79.5 ***	148.2 ***	1.1 ***	73.2 ***	8.07 ***	0.11 ***	92.1 ***	10.04 ***	19.5 ns	2.2 ns	1.4 ***	1.4 ***	2.2 ***	0.7 ***	6.2 ***
Interaction																			
DS * Se	6	0.1 *	8.2 *	9.9 *	2.6 ns	6.7 **	0.06 ***	7.6 ***	1.1 **	0.02 ***	8.8 ***	1.4 **	21.1 ns	22.8 **	7.1 *	1.6 ***	9.7 **	0.1 ***	4.2 *
**Error**	24	0.03	3.1	3.6	1.4	1.4	0.01	0.3	0.2	0.002	0.4	0.3	38.4	4.2	2.5	1.0	1.9	0.01	1.5

*Significant at p ≤ 0.05%; **Significant at p ≤ 0.01%; ***Significant at p ≤ 0.001%; ns at p > 0.05%; Car, carotenoids; Chla, chlorophyll a; Chlb, chlorophyll b; ChlR, chlorophyll ratio; df, degrees of freedom; DS, drought stress; NOB, number of branches; NOL, number of leaves; PBM, plant biomass; PH, plant height; RDW, root dry weight; RFW, root fresh weight; RL, root length; rWC, root water content; Se, selenium; SFW, shoot fresh weight; SDW, shoot dry weight; SL, shoot length; sWC, shoot water content; TChl, total chlorophyll; and TDM, total dry matter.

### 3.2 Physiological characters

**Figure 5:**
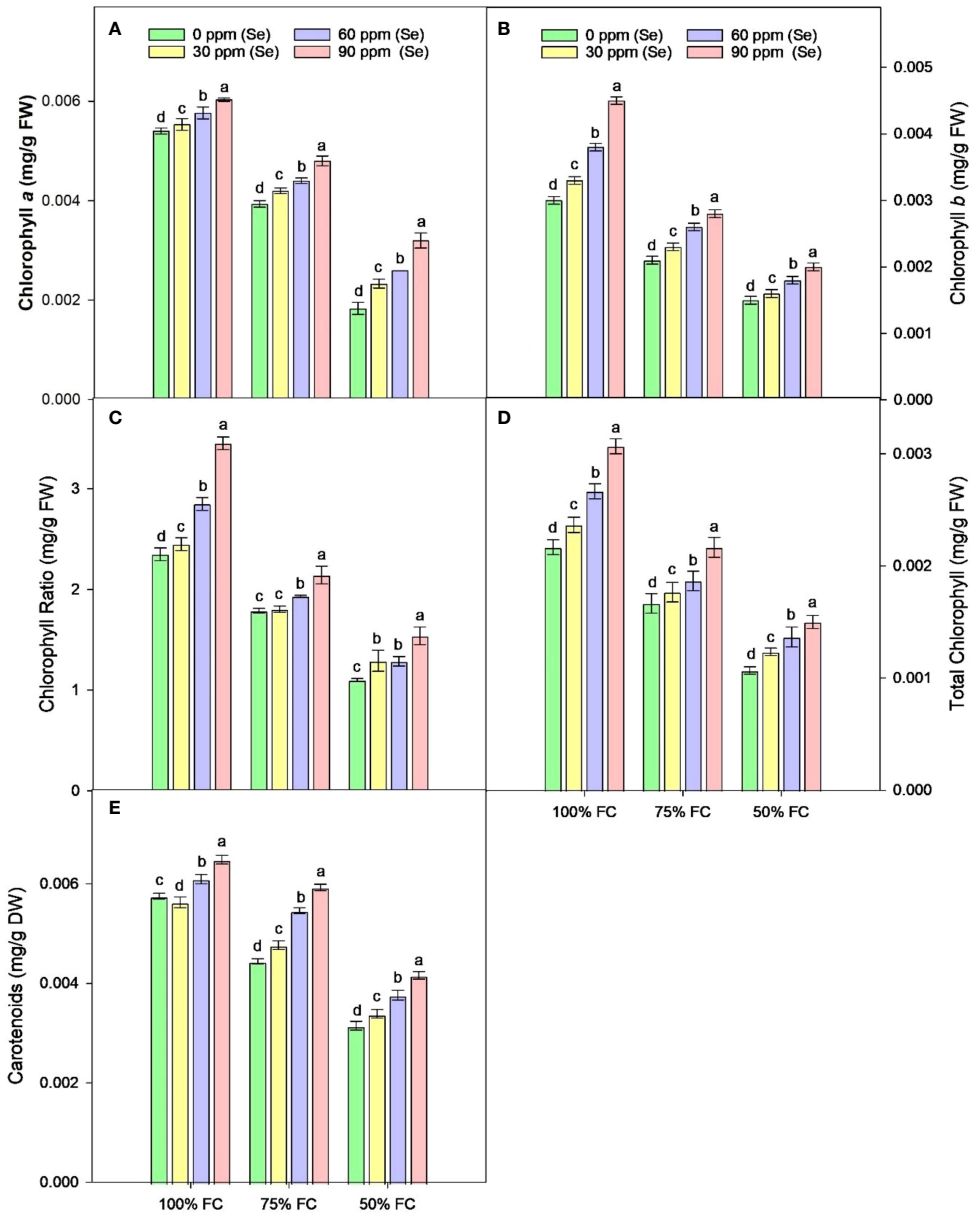
Effect of Se foliar applications on chlorophyll a **(A)**, chlorophyll *b* **(B)**, chlorophyll ratio **(C)**, total chlorophyll **(D)**, and carotenoids **(E)** content of sunflower during drought stress (*n*=3, mean ± SE). The letters (A, B, C, D) represents the respective graph of the different parameters.

Drought stress resulted in the reduction of rWC (2.93%–9.6%) and sWC (2.54%–9.9%) during drought stress compared to control conditions at 50% FC–75% FC. The application of foliar Se (90 ppm) improved rWC and sWC by 0.76%–9.58% and 1.53%–6.57%, respectively, during drought stress (**Figure 4**). The plants subjected to drought conditions experienced a significant (*p* ≈ 0.0184) decrease in the amounts of Chl*a* and Chl*b* and Car in their leaves. Total chlorophyll levels dropped by 27.18% to 49.42% in the leaves of plants that were affected by drought. Similarly, the amount of Car in plants treated with 75% FC and 50% FC dropped by 14.19% and 39.40%, respectively. The amounts of Chl*a*, Chl*b*, and Car in plants improved when Se was applied in controlled or drought conditions. There was an 8.06%–25.81% rise in Chl*a*, 9.09%–40.91% rise in Chl*b*, 9.82%–37.42% rise in TChl, and 5.86%–36.22% rise in chlorophyll ratio (75% FC × Se30, Se60, Se90) compared to respective controls (75% FC × Se0) (**Table 3**; **Figure 5**). When 90 ppm Se was applied to the plants during stress, the amount of Car increased by 3.37% to 24.27%.

### 3.3 Ions, selenium, and protein content

**Figure 6:**
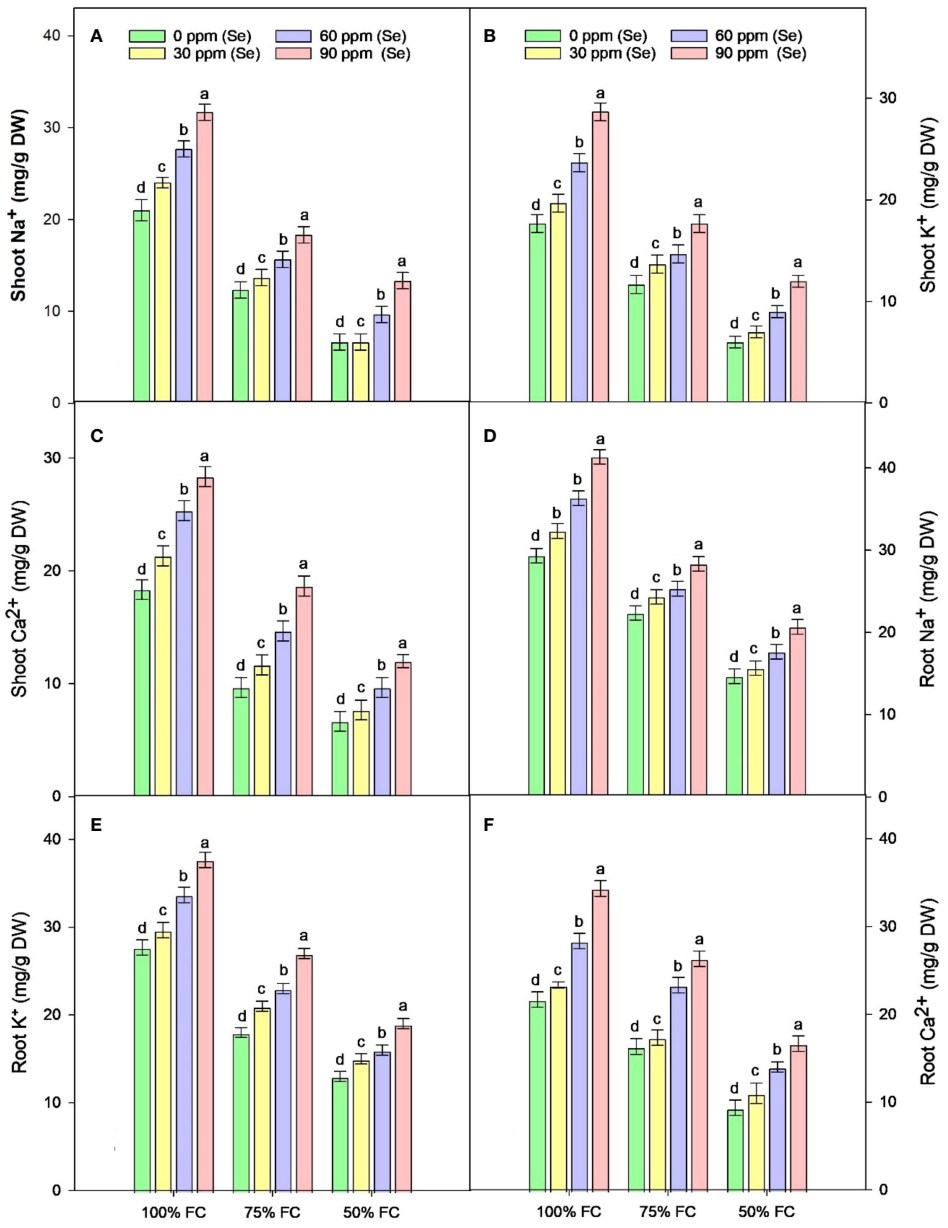
Effect of Se foliar applications on the shoot Na+ **(A)**, shoot K+ **(B)**, shoot Ca2+ **(C)**, root Na+ **(D)**, root K+ **(E)**, and root Ca2+ **(F)** of sunflower during drought stress (*n*=3, mean ± SE). The letters (A, B, C, D) represents the respective graph of the different parameters.

The results of the root and shoot Na^+^, K^+^, and Ca^2+^ ions analysis are presented in **Figure 6** during 75% FC and 50% FC conditions without Se treatments. Na^+^ (24% to 50%), K^+^ (37.02% to 52.99%), and Ca^2+^ (24.58% to 56.97%) ion concentrations gradually decreased in roots. With further increase in Se treatment levels from 30 ppm to 90 ppm to the pots experiencing severe drought stress (50% FC), Na^+^ (6.82%–40.94%), K^+^ (15.38%–46.15%), and Ca^2+^ (17.86%–78.57%) ion concentrations gradually increased in roots. The highest concentration of Na^+^, K^+^, and Ca^2+^ was measured at 90 ppm Se with 75% FC during drought stress with an increase of 26.88%, 50%, and 61.29%, respectively, as compared to control (75% FC × Se0).

Analysis of ion content in shoots revealed that Na^+^, K^+^, and Ca^2+^ ion concentrations decline with the elevation of drought conditions. The minimum decline in concentration of Na^+^, K^+^, and Ca^2+^ ions was observed at 50% FC with a range of 65.09%, 61.91%, and 61.45%, respectively (**Figure 6**). During drought stress, Se application at different concentrations caused a further increase in ion concentrations in shoots compared to the control without Se. The highest Na^+^, K^+^, and Ca^2+^ ion content in shoots during drought stress was determined at 75% FC with 90 ppm Se treatment with an increase of 48.66%, 51.43%, and 93.14%, respectively (**Table 3**). The lowest amount of Na^+^, K^+^, and Ca^2+^ ions was observed at 50% FC × Se30 by 0.1%, 16.67%, and 15.00%, respectively.

**Figure 7:**
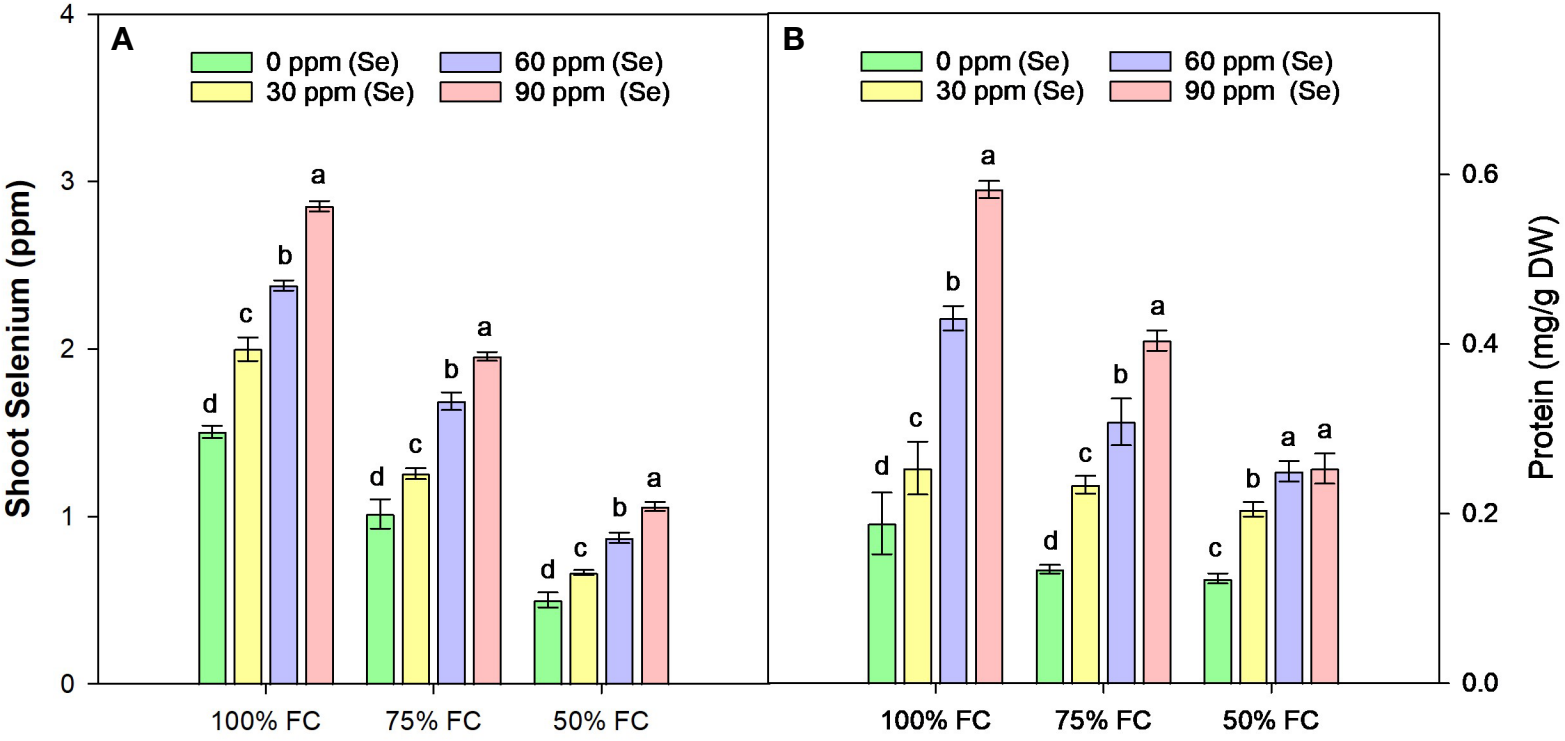
Effect of Se foliar applications on the shoot selenium **(A)** and total soluble protein **(B)** content of sunflower during drought stress (*n*=3, mean ± SE). The letters (A, B, C, D) represents the respective graph of the different parameters.

In sunflower with 100% FC × Se0, the average Se content in shoot was 1.5 ppm, which decreased by 64.46% plants with 50% FC (**Figure 7A**). The ability of the sunflower to absorb Se was thus greatly influenced by the water availability. One possible explanation for the Se found in the untreated plants’ shoots is that the soil in which the sunflowers were grown naturally contains Se. The treatment with sodium selenate increased the Se content of shoots, with average increases of 23.62%, 66.23%, and 92.32% at 30, 60, and 90 ppm Se treatments, respectively, during 75% FC drought stress.

Drought stress reduced Pro (**Figure 7B**) by 34.41% as compared to normal water supply. The lowest amount of Pro was observed at 50% FC × Se30, with a reduction of 104.85 as compared to control. The highest Pro (0.58 mg/g FW) content was observed in plants treated with 90 ppm Se during 100% FC. Nonetheless, there was a very strong interaction between the availability of Se and drought stress levels (**Table 4**). The water stressed plants (75% FC) with Se (90 ppm) had the highest Pro (0.40 mg/g FW) accumulation, which was 200.34% higher than the control treatments (75% FC × Se0).

**Table 4:** The statistical analysis (ANOVA) showing mean square values of Se applications on biochemical and ionic attributes of sunflower during drought stress.

Source	df	RNa	RK	RK	SNa	SK	SCa	Se	TP	AsA	Flv	Ant	Pro	POD	SOD	CAT	GB	H_2_O_2_
Main effects																		
DS	2	939.7 ***	820.2 ***	820.2 ***	893.7 ***	585.3 ***	641.3 ***	5.9 ***	796.6 ***	0.004 ***	13 ***	45.9 ***	0.07 ***	74.14 ***	43.72 ***	0.13 ***	1.3 ***	0.005 ***
Se	3	106.2 ***	113.7 ***	113.7 ***	106.3 ***	99.3 ***	113.1 ***	1.5 ***	167.5 ***	1.7 ***	4.7 ***	18.5 ***	0.12 ***	83.4 ***	34.1 ***	0.01 ***	3.1 ***	0.001 ***
Interaction																		
DS * Se	6	7.2 *	3.41 ns	3.41 ns	4.1 ns	5.5 *	3.8 ns	0.08 ***	3.73 *	7.4 **	0.14 *	1.72 ***	0.013 ***	0.6 **	0.3 *	3.9 **	1.2 *	3.81 ns
Error	24	2.3	1.4	1.4	2.3	1.9	2.2	0.006	1.46	1.6	0.04	0.1	0.001	0.12	0.11	9.76	4.7	1.8

*Significant at p ≤ 0.05%; **Significant at p ≤ 0.01%; ***Significant at p ≤ 0.001%; ns at p > 0.05%; AsA, ascorbic acid; Ant, anthocyanin; CAT, catalase; df, degrees of freedom; DS, drought stress; Flv, flavonoids; GB, glycine betaine; H_2_O_2_, hydrogen peroxide; POD, peroxidase; Pro, total soluble protein; RCa, root calcium; RK, root potassium; RNa, root sodium; SCa, shoot calcium; Se, selenium; SK, shoot potassium; SNa, shoot sodium; SOD, superoxide dismutase; SSe, shoot selenium; and TP, total phenolics.

### 3.4 Secondary metabolites

**Figure 8:**
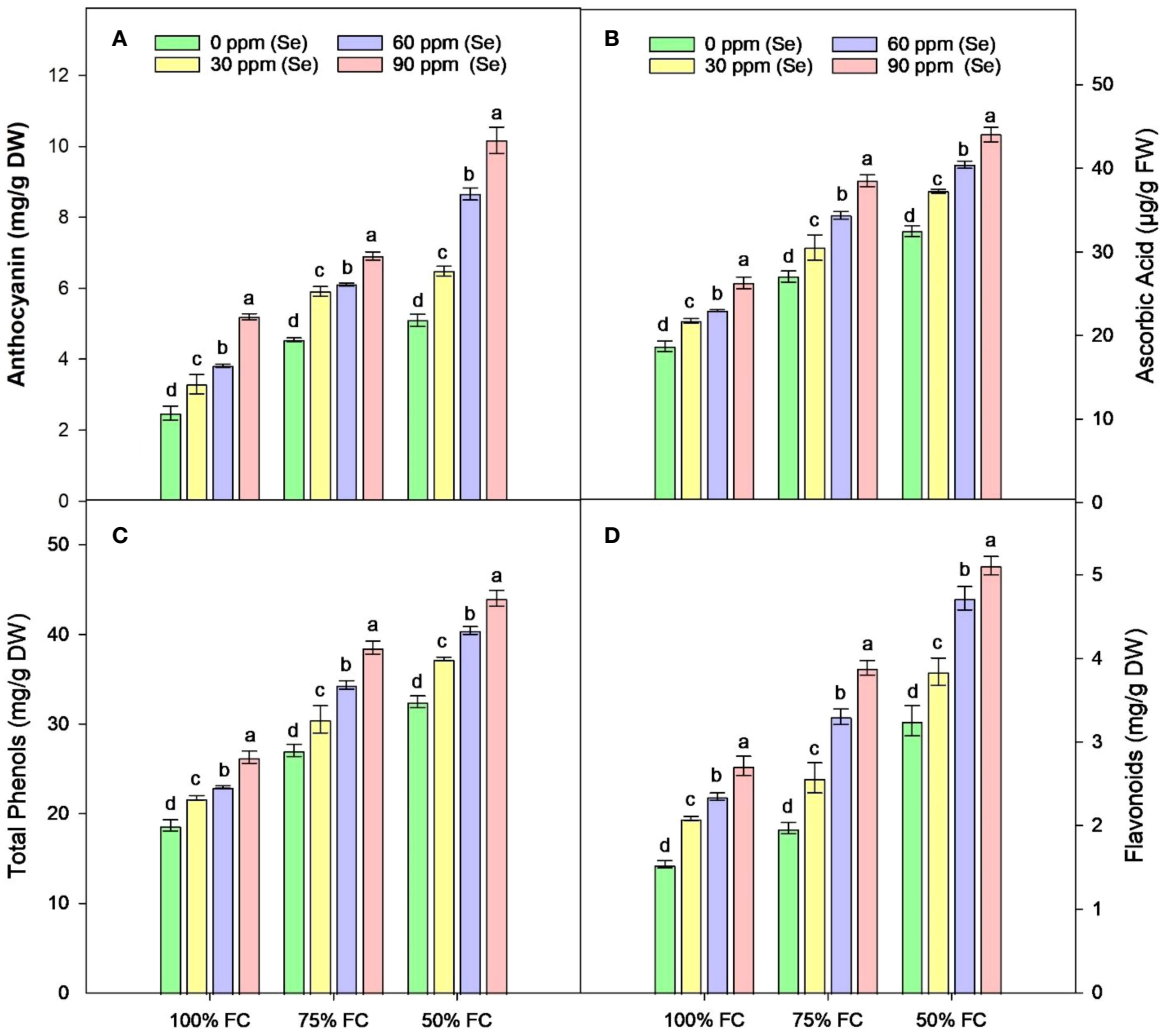
Effect of Se foliar applications on the anthocyanin **(A)**, ascorbic acid **(B)**, total phenols **(C)**, and flavonoids **(D)** accumulation of sunflower during drought stress (*n*=3, mean ± SE). The letters (A, B, C, D) represents the respective graph of the different parameters.

The drought stress greatly increased the amounts of AsA, TP, Flv, and anthocyanins (Ant) in plants. It was observed that the amounts of TP, Flv, Ant, and AsA rose by 41.79%, 48.77%, 51.33%, and 57.06%, respectively, compared to the control plants. Using Se and drought together led to even higher levels of TP, Flv, and AsA in plants with 100% FC. In contrast, plants that were treated with 75% FC × Se90 showed increases of 24.66% in AsA, 42.43% in TP, 97.76% in Flv, and 51.85% in Ant compared to plants treated with 75% FC × Se0 (**Figure 8**). There was a 35.65% increase in TP and a 57.35% rise in Flv when 90 ppm of Se was applied during 50% FC drought stress. Meanwhile, Ant showed the best response, with a 99.41% rise in their content (**Table 4**) under the 50% FC × Se90 treatment.

### 3.5 Enzymatic antioxidants

**Figure 9:**
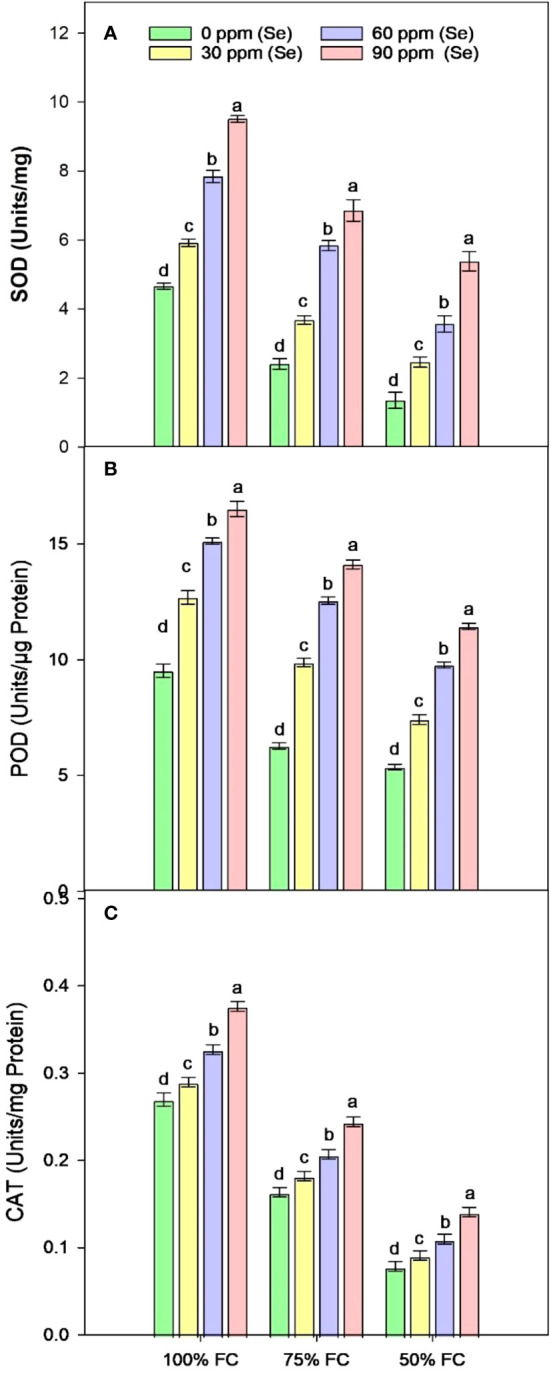
Effect of Se foliar applications on SOD **(A)**, POD **(B)**, and CAT **(C)** activity of sunflower during drought stress (*n*=3, mean ± SE). The letters (A, B, C, D) represents the respective graph of the different parameters.

Sunflower plants grown under various drought conditions exhibited lower levels of SOD, CAT, and POD activities. CAT activities decreased by 77.75%, SOD activities decreased by 54.30%, and POD activities decreased by 43.68% compared to the control plants during drought stress (**Figure 9**). Enzyme activities increased after Se foliar application, indicating the recovery of the antioxidant defense system. Relative to 100% FC plants, increases of 26.98%–103.83% for SOD, 33.18%–73.03% for POD, and 7.42%–39.63% for CAT were observed under application of 90 ppm Se (**Table 4**). However, in plants that were severely stressed by drought (50% FC), the application of 90 ppm Se positively enhanced enzyme activities, increasing CAT activity by 78.93%, POD activity by 113.33%, and SOD activity by 298.89%.

### 3.6 Osmoprotectants

**Figure 10:**
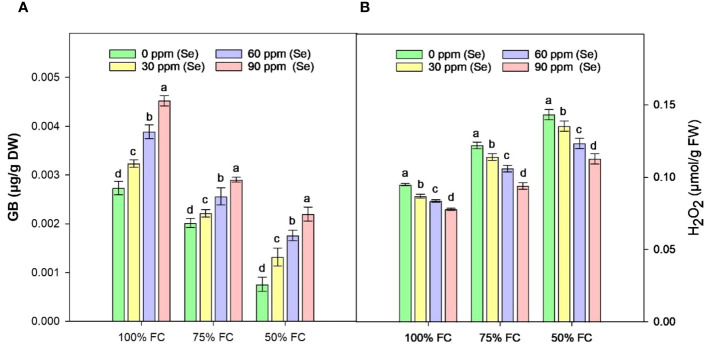
Effect of Se foliar applications on the GB **(A)** and H2O2 **(B)** content of sunflower during drought stress (*n*=3, mean ± SE). The letters (A, B, C, D) represents the respective graph of the different parameters.

**Figure 10A** shows that the highest GB content was 65.91% under controlled conditions (100% FC × Se90). When irrigation was reduced to 75% FC, the amount of GB decreased by 33.15% and it decreased by 57.10% under 50% FC drought stress compared to 100% FC. The lowest amount of GB was observed at 50% FC × Se30 with a reduction of 73.68% as compared to control (**Table 4**). However, the highest increase in GB during drought stress occurred at 75% FC × Se90 conditions compared with the other drought conditions. As illustrated in **Figure 10A**, there was a significant difference between Se levels in the form of GB; the amount of GB that increased at 75% FC (90 ppm Se) was 43.56% less than the GB content at 50% FC (90 ppm Se), which was equal to 189.47%. Nevertheless, the concentration of 90 ppm of Se proved most significant for osmoregulation, causing GB content to increase to 0.0029 µg/g DW.

### 3.7 Reactive oxygen species

The contents of H_2_O_2_ in the leaves of sunflower exhibited a significant rise in response to drought stress (**Figure 10B**). Increases of 15.23% and 33.30% in H_2_O_2_ content were observed at 75% FC and 50% FC, respectively, compared to control plants. However, Se application lowered these H_2_O_2_ levels. In combination with 75% FC × Se30, Se60, and Se90, a marked reduction of 6.56%, 13.11%, and 23.03%, respectively, was observed as compared to the 75% FC × Se0 (**Table 4**). The highest decrease in H_2_O_2_ (0.11 µmol/g FW) was observed in plants treated with 90 ppm Se application during 50% FC, which represented approximately a 21.30% reduction compared to 50% FC × Se0.

### 3.8 Heatmap

**Figure 11:**
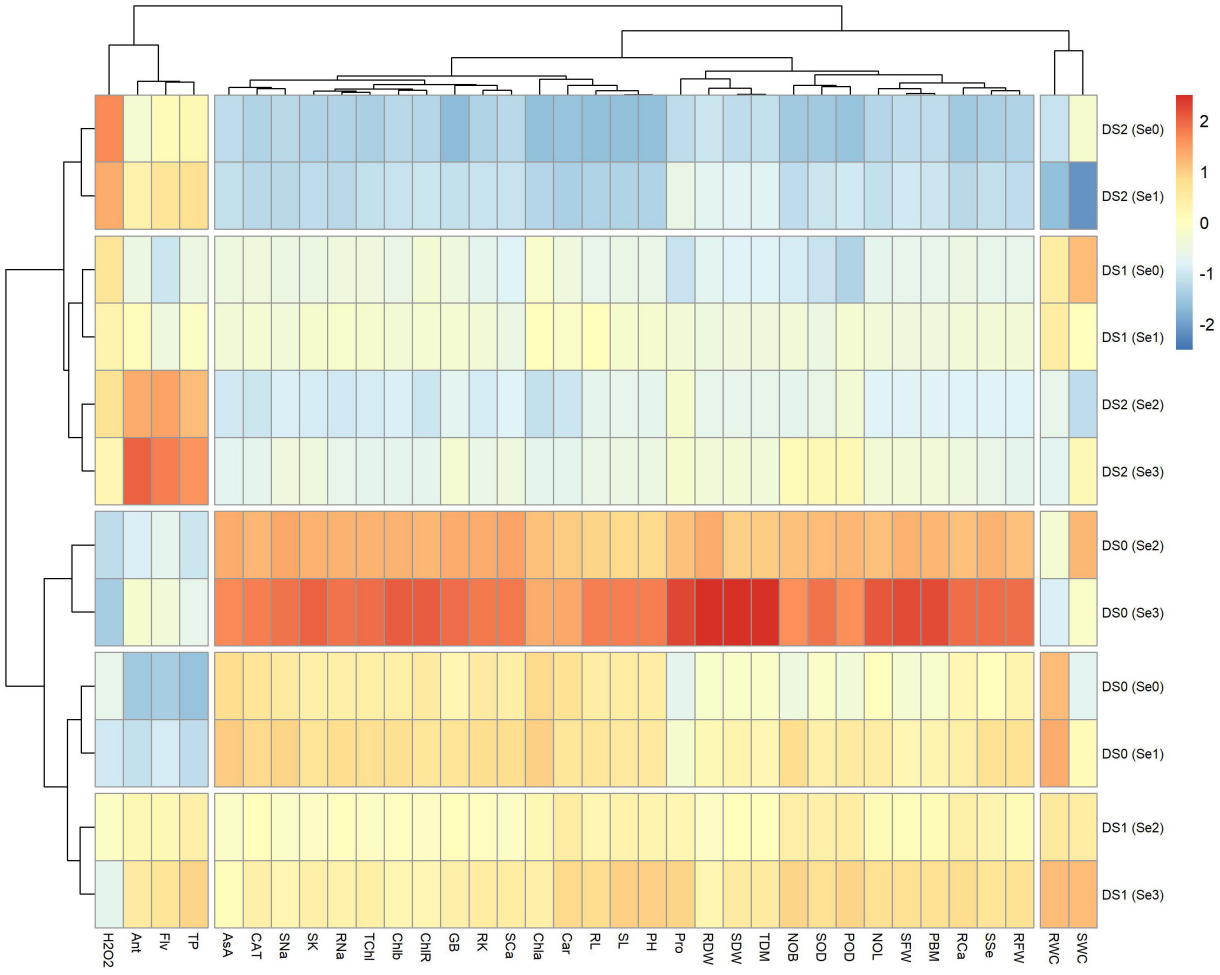
Heatmap showing interaction of morpho-physiological, biochemical, enzymatic, and nutrient attributes of sunflower during drought stress. Where the DS1 (100% FC), DS2 (75% FC), DS3 (50% FC), Se0 (0 ppm), Se1 (30 ppm), Se2 (60 ppm), Se3 (90 ppm), AA (Ascorbic acid), Ant (Anthocyanin), Car (Carotenoids), CAT (Catalase), Chla (Chlorophyll a), Chlb (Chlorophyll b), ChlR (Chlorophyll ratio), Flv (Flavonoids), GB (Glycine betaine), H2O2 (Hydrogen peroxide), NOB (Number of branches), NOL (Number of leaves), PBM (Plant biomass), PH (Plant height), POD (Peroxidase), Pro (Total soluble Protein), RCa (Root calcium), RDW (Root dry weight), RFW (Root fresh weight), RL (Root length), RK (Root potassium), rWC (root water content), RNa (Root sodium), SCa (Shoot calcium), SFW (Shoot fresh weight), SK (Shoot potassium), SL (Shoot length), sWC (shoot water content), SNa (Shoot sodium), SOD (Superoxide dismutase), SSe (Shoot selenium), TChl (Total chlorophyll), TDM (Total dry matter), and TP (Total phenolics).

The heatmap (**Figure 11**) was constructed to analyze the relationships among morpho-physiological and biochemical parameters under different drought stress levels (DS0 = 100% FC, DS1 = 75% FC, and DS2 = 50% FC) and exogenously applied selenium (Se0 = 0 ppm, Se1 = 30 ppm, Se2 = 60 ppm, and Se3 = 90 ppm). The main cluster showed three distinct subclusters. In the first subcluster, there was a positive grouping among Ant, Flv, and TP at DS2 (Se2) and DS2 (Se3), while a negative association was observed at DS0 (Se0) and DS0 (Se1). Similarly, hydrogen peroxide showed a positive association at DS2 (Se0) and DS2 (Se1) while exhibiting a negative association at DS0 (Se2) and DS0 (Se3). In the second subcluster, plant ionic (SNa, RNa, SCa, SK, and RK), photosynthetic (TCHL, Chl*b*, and CHLR), and biochemical (AsA, CAT, and GB) showed a highly positive association at DS0 (Se3) and DS0 (Se2), while showing negative grouping at DS2 (Se0), DS2 (Se1), and DS2 (Se2). Similarly, in the same group, Pro, RDW, SDW, TDM, NOL, SFW, and PBM showed a highly positive association at DS0 (Se3), but a strong negative association at DS2 (Se0). In the third subgroup, rWC revealed a positive association at DS1 (Se3), DS0 (Se0), and DS0 (Se1), while sWC displayed a positive association at DS1 (Se3), Ds0 (Se2), and DS1 (Se0). Both rWC and sWC showed a negative association at DS2 (Se1).

### 3.9 Principal component analysis

**Figure 12:**
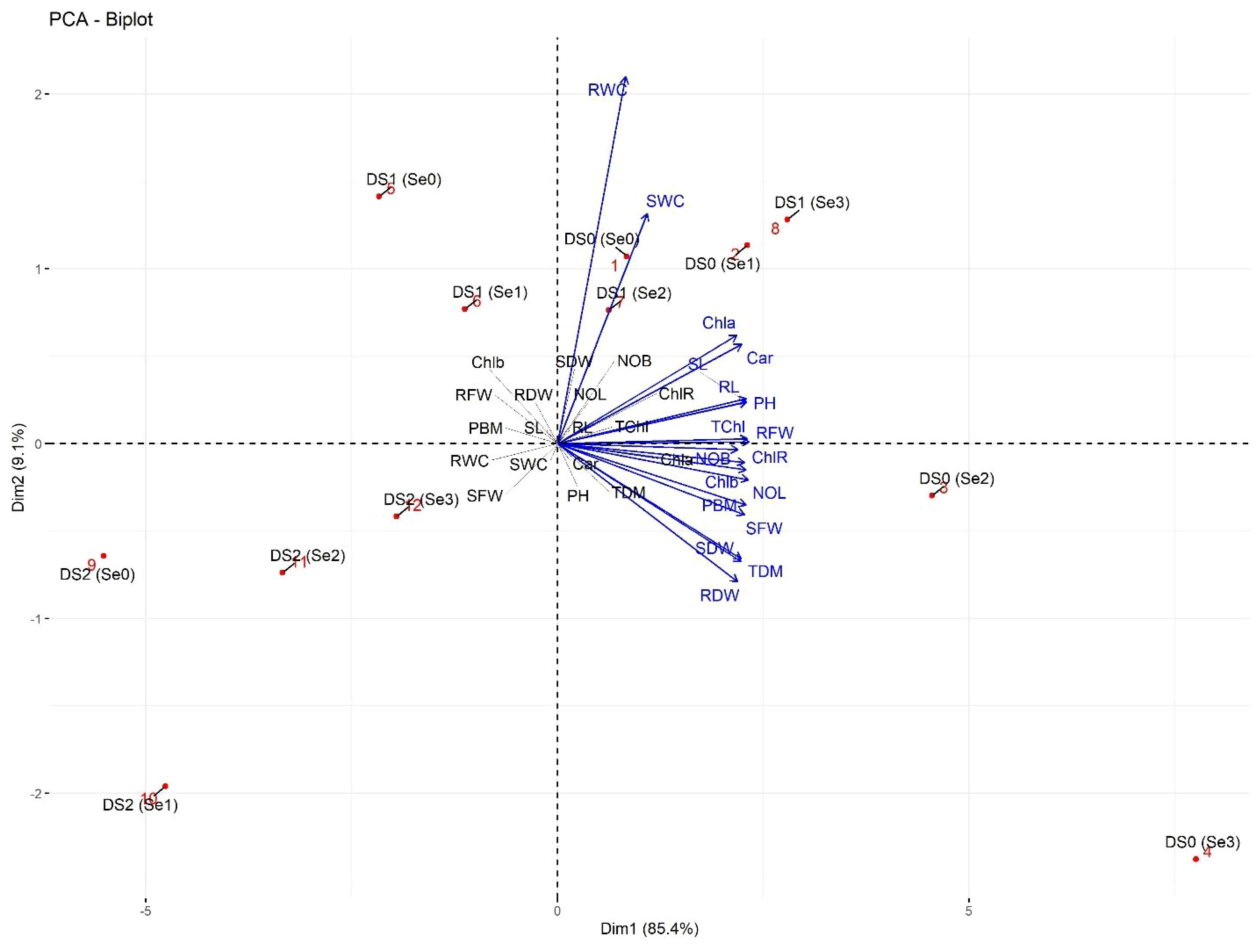
PCA-Biplot of morpho-physiological attributes of sunflower during drought stress. Where the Car (Carotenoids), Chla (Chlorophyll a), Chlb (Chlorophyll b), ChlR (Chlorophyll ratio), DS1 (100% FC), DS2 (75% FC), DS3 (50% FC), NOB (Number of branches), NOL (Number of leaves), PBM (Plant biomass), PH (Plant height), RDW (Root dry weight), RFW (Root fresh weight), RL (Root length), rWC (root water content), SDW (Shoot dry weight), Se0 (0 ppm), Se1 (30 ppm), Se2 (60 ppm), Se3 (90 ppm), SFW (Shoot fresh weight), SL (Shoot length), sWC (shoot water content), TChl (Total chlorophyll), and TDM (Total dry matter).

The PCA biplot of morpho-physiological parameters showed two distinct clusters (**Figure 12**). In the first subcluster, rWC and sWC were strongly linked to each other at DS0 (Se0) and DS1 (Se2). Similarly, plant photosynthetic traits including Chl*a*, Car, and TChl were strongly associated with RL, SL, RFW, and PH at DS0 (Se1) and DS1 (Se3). Secondly, the number of branches and leaves, as well as PBM, showed a positive association with SFW, SDW, RDW, and TDM at DS0 (Se2).

**Figure 13:**
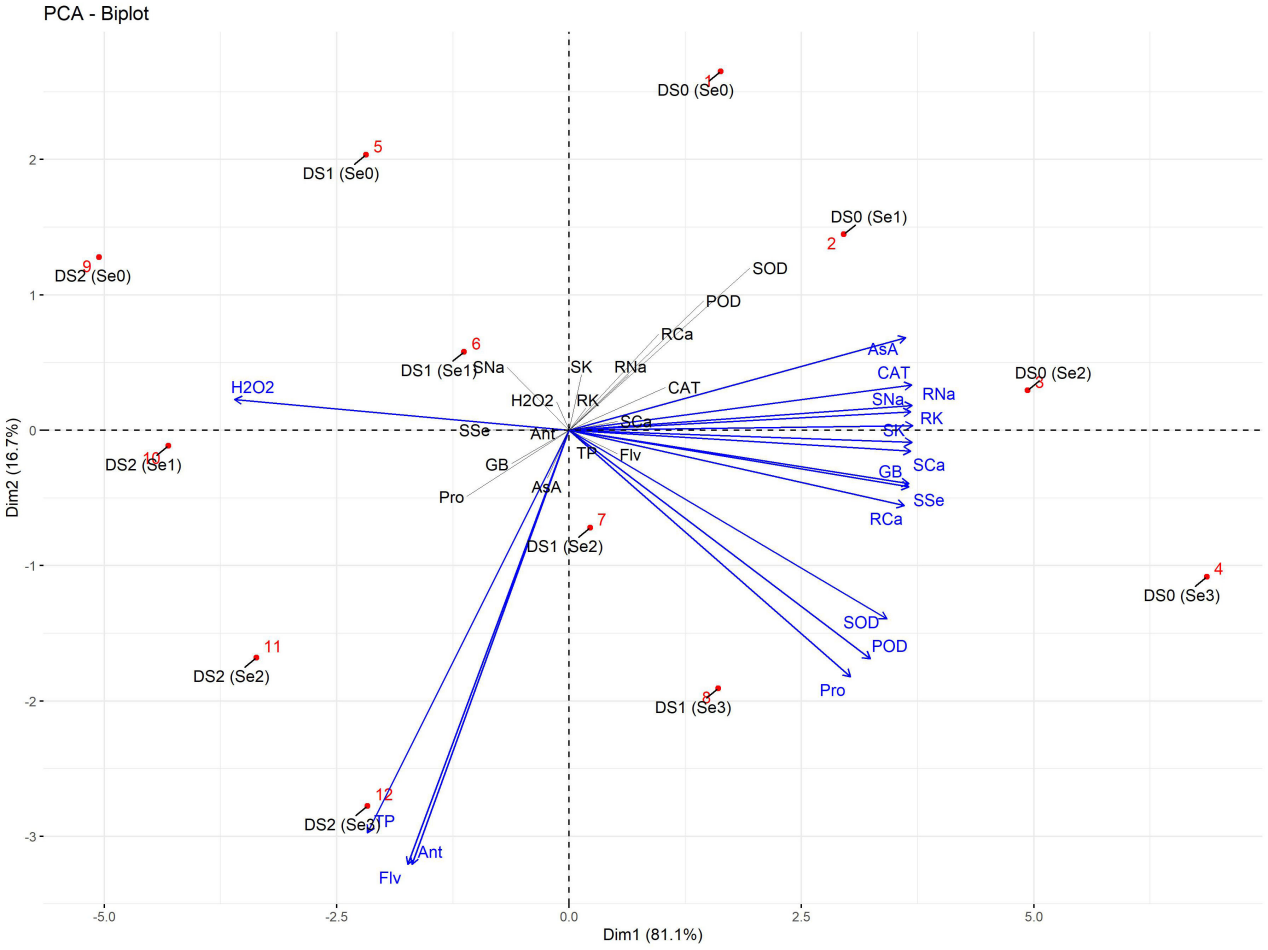
PCA-Biplot of biochemical, enzymatic, and nutrient attributes of sunflower during drought stress. Where the AA (Ascorbic acid), Ant (Anthocyanin), CAT (Catalase), DS1 (100% FC), DS2 (75% FC), DS3 (50% FC), Flv (Flavonoids), GB (Glycine betaine), H2O2 (Hydrogen peroxide), POD (Peroxidase), Pro (Total soluble protein), RCa (Root calcium), RK (Root potassium), RNa (Root sodium), SCa (Shoot calcium), Se0 (0 ppm), Se1 (30 ppm), Se2 (60 ppm), Se3 (90 ppm), SK (Shoot potassium), SNa (Shoot sodium), SOD (Superoxide dismutase), SSe (Shoot selenium), and TP (Total phenolics).

The PCA biplot of biochemical, enzymatic, and nutrient attributes showed four isolated clusters (**Figure 13**). H_2_O_2_ was strongly linked at DS2 (Se1), while AsA and CAT were positively linked with root and shoot Na^+^ at DS0 (Se2). Similarly, TP was strongly linked to Flv and Ant at DS2 (Se3). Additionally, SOD POD, GB, and Pro showed a strong association with root Ca^2+^ and shoot Ca^2+^ and K^+^ linked at DS1 (Se3).

### 3.10 Correlation

**Figure 14:**
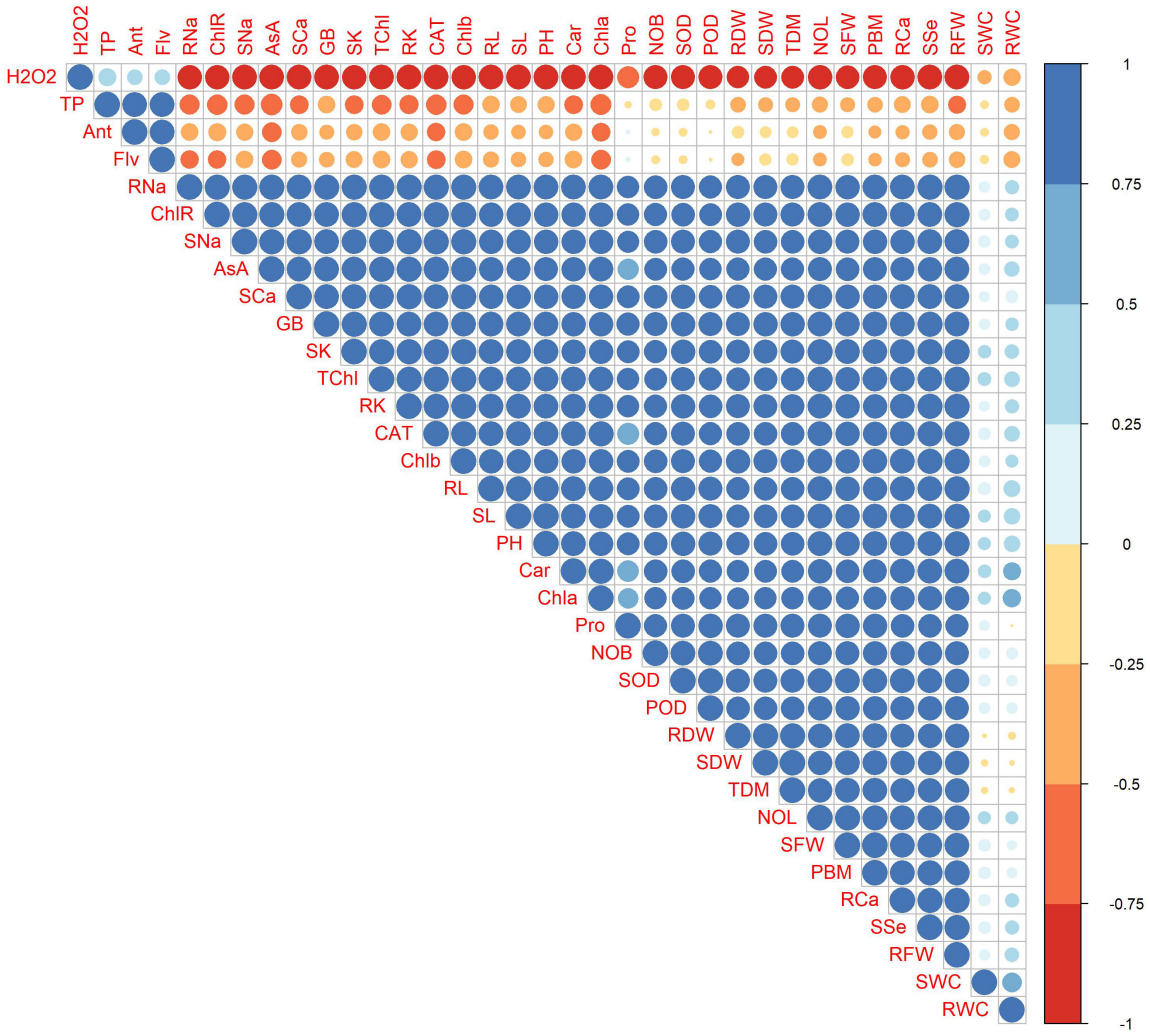
Correlation plot of morpho-physiological, biochemical, enzymatic, and nutrient attributes of sunflower during drought stress, where the AA (Ascorbic acid), Ant (Anthocyanin), Car (Carotenoids), CAT (Catalase), Chla (Chlorophyll a), Chlb (Chlorophyll b), ChlR (Chlorophyll ratio), Flv (Flavonoids), GB (Glycine betaine), H2O2 (Hydrogen peroxide), NOB (Number of branches), NOL (Number of leaves), PBM (Plant biomass), PH (Plant height), POD (Peroxidase), Pro (Total soluble protein), RCa (Root calcium), RDW (Root dry weight), RFW (Root fresh weight), RK (Root potassium), RL (Root length), RNa (Root sodium), rWC (root water content), SCa (Shoot calcium), SDW (Shoot dry weight), SFW (Shoot fresh weight), SK (Shoot potassium), SL (Shoot length), SNa (Shoot sodium), sWC (shoot water content), SOD (Superoxide dismutase), SSe (Shoot selenium), TChl (Total chlorophyll), TDM (Total dry matter), and TP (Total phenolics).

Correlations between morpho-physiological and biochemical traits are exhibited in **Figure 14**. H_2_O_2_ showed a positive correlation with TP, Ant, and Flv, while exhibiting a strong negative correlation with growth parameters (RL, SL, PH, RFW, RDW, SFW, SDW, NOL, and NOB), ionic contents (root and shoot Ca^2+^, K^+^, Na^+^, and SSe ions), photosynthetic parameters (Chl*a*, Chl*b*, Car, TChl, and ChlR), and AsA, GB, CAT, and Pro. Root Na^+^, ChlR, AsA, CAT, and Chl*a* showed strong negative correlation with H_2_O_2_, AsA, Ant, and Flv, while rWC and sWC are positively associated with factors such as RFW, SFW, Pro, and TDM. Conversely, they are negatively correlated with H_2_O_2_, indicating that lower oxidative stress is linked to higher water content. This suggests that maintaining cellular health and managing stress responses are crucial for optimal water retention in plants. Overall, plant photosynthetic pigments and ionic contents were positively correlated with growth parameters.

## 4 Discussion

Plants grown in pots that were not receiving sufficient water exhibited significantly shorter roots and shoots, as well as reduced fresh and dry weights. Shoots showed fewer signs of dwarfism compared to roots, likely due to their direct exposure to the drought and other environmental factors. According to Zia et al. (2021), drought conditions may prolong the cell cycle period, negatively affecting both cell division and cell extension, thereby impairing root and shoot growth. Inadequate root growth can reduce nutrient accessibility for shoots, consequently leading to poor shoot development.

According to an earlier study by Ahmad et al. (2020) on *Brassica napus* L. and *Camelina sativa* L., roots and plants exhibited reduced growth when they were exposed to drought. Nevertheless, Se supplementation in our study helped drought-stressed plants grow better. This improvement could be due to Se’s beneficial effects on carbohydrate metabolism, which supports plant growth. Furthermore, our research revealed that Se enhanced overall photosynthetic efficiency, leading to increased carbohydrate production and ultimately better growth (Wang et al., 2023b). Previous studies have also observed a comparable enhancement in growth when applying Se to *Oryza sativa* L (Yin et al., 2019), *Solanum tuberosum* L (Zhang et al., 2019), and *Lactuca sativa* plants (Shalaby et al., 2017) under abiotic stress.

We have shown that sunflowers retain a high level of rWC and sWC even in drought conditions. The observed reduction in rWC and sWC in this experiment can be attributed to a cellular water deficit resulting from a transpiration rate that exceeds water intake. The impact of Se on sWC did not show a significant effect (*p* = 0.538) in reducing drought stress. However, its influence on rWC was significantly different (*p* ≈ 0.22) from sWC. Song et al. (2024) observed that Se improved water content and reduced transpirational loss in tomato. Bocchini et al. (2018) examined that the Se supplementation improved the water balance in *Zea mays* L. when exposed to drought and high salinity stress. Nazim et al. (2023) observed that the application of Se enhanced the levels of soluble proteins and free amino acids, improved water usage efficiency, and enhanced many physiological parameters in sunflower plants. Comparable results have been shown in sweet potato (Huang et al., 2020), rice (Ghouri et al., 2022), wheat (Sardari et al., 2022), and tobacco (Han et al., 2022). According to Fan et al. (2022), Se enhanced root development and growth while decreasing transpiration rate during water stress and increasing water content. rWC was found to positively correlate with PBM, amount of chlorophyll, RL, and antioxidant activity in the present study.

This study shows that water stress lowers the amount of Chl and Car in plants. However, supplementing sunflowers with Se lowered the adverse effects of drought conditions. The drop in leaf carotenoid level during drought could be because oxidative stress causes more chlorophyll degradation or reduces chlorophyll biosynthesis (Dhami and Cazzonelli, 2020). A common effect of drought stress is a drop in photosynthetic pigments. It is a frequently observed phenomena that the decrease in photosynthetic pigments under drought stress serves as a sensitive indicator of the metabolic condition of cells (Li et al., 2020).

It was found by Mathobo et al. (2017) that drought stress reduced the amount of Chl*a* and Chl*b* in leaves. On the other hand, adding Se to the pots caused the amount of carotenoids to increase. Drought-stressed sunflower plants accumulated more Chl and Car when 5 µM Se (selenate) was applied, as reported by Hawrylak-Nowak et al. (2015). According to Liu et al. (2023), Se helps tomato plants deal with drought stress by controlling the antioxidant defense mechanisms within the chloroplasts linked to an increase in the photochemical activity of photosystem-II (PS-II).

In this study, drought stress greatly reduced the number of ions like Na^+^, K^+^, and Ca^2+^ in the sunflower roots and shoots (**Figure 6**). Se regulates the uptake and transfer of these essential ions, and it may help to activate antioxidants that lower ROS levels, enabling plants to better survive within stress conditions (Azeem et al., 2023). Adding Se to the plant improved the levels of Na^+^ and K^+^, but it had a lower effect on the levels of Ca^2+^ in the roots and shoots compared to other ions. These results agree with Zembala et al. (2010), who observed a gradual rise in the distribution of macro- and microelement in wheat plants through exogenous Se treatment under cadmium stress. Rasool et al. (2023) found that Se regulates the ion channels to prevent ion toxicity and maintain a steady K+/Na+ ratio in the plant.

When Se combined with other essential micronutrients like silicon (Si) (Ghouri et al., 2022) and molybdenum (Mo) (Al-Jaberi et al., 2019), or macronutrients like chloride (Cl^−^) (Franco-Navarro et al., 2021), magnesium (Mg^2+^) (Ahmed et al., 2023), and nitrate (NO_3_^−^) (Lei et al., 2018), it can further improve drought tolerance and plant productivity. Si strengthens cell walls and reduces transpiration, Mg^2+^ is vital for chlorophyll production and enzyme activation, and K maintains osmotic balance and enzyme activation, enhancing water retention and stress resistance. Mo supports nitrogen metabolism and stress response, while NO_3_^−^ is crucial for protein synthesis and growth, supporting plant development during drought stress (Singhal et al., 2023). Recent studies have also proposed Cl^−^ as a beneficial macronutrient that, like Se, contributes to improving drought tolerance by influencing leaf cell size, stomatal conductance, plant water conservation, nutrient use efficiency (NUE), and overall drought tolerance (Franco-Navarro et al., 2016; Rosales et al., 2020; Franco-Navarro et al., 2021). Future research should focus on the integrated application of these nutrients with Se, their molecular mechanisms, and field trials to optimize nutrient management strategies under drought conditions.

The K^+^ is a primary nutrient in drought-stressed plants, aiding in osmoregulation, water uptake, cell expansion, stomatal opening, transpiration flow, and photosynthesis. Jiang et al. (2017) observed a rise in *Z. mays* L. K^+^ levels when supplemented with Se. Furthermore, compared to organic metabolites, it is a cost-effective osmoprotectant. Lu et al. (2021) note that Na is an effective osmoprotectant that is typically accumulated within the vacuole to reduce cell injuries. Concentrations of Na and K are good indicators of a plant’s ability to handle drought. For instance, Haghighi et al. (2016) investigated the variation in internal CO_2_ caused by the effect of Se on the uptake of K^+^, which alters stomatal opening and subsequently causes internal CO_2_ to fluctuate depending on the quantity of Se used.

Our results revealed that adding supplementary Se had significant effects on the concentrations of Na^+^, K^+^, and Ca^2+^ in sunflower. Se might affect how well nutrients are absorbed because it influences ion accumulation in plant cells by interacting with the plasmalemma and cellular metabolic processes. Se may change the permeability rate of plasmatic membrane for some ions, which could alter the circulation of other ions within plants. Wu et al. (2014) discovered that during drought stress, the contents of Na^+^ and K^+^ in three different varieties of *Beta vulgaris* L. either decreased or did not change during drought stress, which is in contradiction to our findings.

When we applied Se to the leaves of plants under different types of drought conditions, the treatment with 90 ppm Se resulted in the highest concentration of Se in the shoots compared to the control (**Figure 7A**). In 2017, Mroczek-Zdyrska et al. (2017) found that the addition of Se to the soil increased the amount of Se in *Vicia faba* L. plants and found a strong correlation between the amount of Se in shoots and glutathione peroxidase (GSH-Px) activity. Our findings are comparable to those of Yadav et al. (2023) in wheat; they suggested that plants can accumulate excessive Se when there is less moisture in the soil. Although applying Se to the leaves of stressed plants slightly increased the amount of Se in their stems, this could be because Se has higher antioxidant activity. As Se is more readily available in low-precipitation conditions, our results are in line with the findings of Nawaz et al. (2015) in wheat, who proposed that soil moisture influences the availability of Se to plants. According to Mounicou et al. (2006), sunflowers are not Se accumulators, meaning they cannot store a large amount of the element in their tissues. However, foliar spray was an effective way for the plant to absorb Se.

Water stress conditions led to an increase in the uptake and accumulation of Se. The highest amount of Se was found in plants that were both water-stressed and received supplementary Se through foliar application. The observed increase may be attributed to the higher antioxidant activity exhibited by Se in comparison to the control (Zahedi et al., 2021). Additionally, Mroczek-Zdyrska et al. (2017) observed that soil Se fertilization raised the concentration of Se in *V. faba* L. seedlings and reported a strong positive association between SSe concentration and GSH-Px activity. These findings align with those of Yadav et al. (2023) in wheat, who suggested that plant availability of Se is influenced by soil moisture, with plants having more accessibility to Se at a lower precipitation rate in the surroundings.

It is normal for plants to accumulate secondary metabolites like Ant, AA, TP, and Flv when they are subjected to unfavorable conditions. The rise in secondary metabolites in sunflower plants during drought is because these substances may assist with reduction of membrane lipid peroxidation and eliminate ROS (Rasheed et al., 2022). Phenolic substances work as an intermediary ROS acceptor in the cell and transform into phenoxyl radicals to protect cells from damage in stressful situations like drought (Hasanuzzaman et al., 2020). Phenylalanine, a precursor to phenolic compounds, is among the many amino acids that are positively impacted by selenium. Se also increases polyphenols by stimulating the phenylpropanoid biosynthesis pathway (Wang et al., 2023a). Se treatment leads to a noticeable increase in total phenol content in *O. sativa* L (Chauhan et al., 2017). and *Malus domestica* (Groth et al., 2020). In this context, these responses can be seen as important indicators of specific signals, changes in the secondary metabolism, and activation of defense-related responses, suggesting that Se may help plants become highly tolerant to drought.

This study also found that applying Se led to an increase in Pro content (**Figure 7B**). This finding is similar to that of Habibi (2017), who found that applying Se to sunflower plants during drought stress led to a significant rise in Pro level. The drop (47.24%) in Pro is because the rate of protein formation slows down and more proteins break down during drought stress, which is needed to produce low-molecular-weight osmolytes for osmotic adjustment, also known as osmoregulation (Michaletti et al., 2018). Higher protease activities may cause the concentration of Pro decrease in water-stressed seedlings. This is consistent with research by Bocchini et al. (2018) and Luo et al. (2021), which found that plants lose water and develop low-molecular-weight Pro instead of high-molecular-weight Pro. They also concluded that biochemical factors such as total protein, soluble sugars, and free amino acids all change when plants are under water-deficient conditions. In contrast to our findings, Mohammed (2018) found that increased nitrate reductase activity and free amino acids were responsible for the rise in Pro levels in sunflower plants treated with Se.

During this study, GB showed a wide range of responses, and the lack of water had a considerable impact on it. Most of the GB was found in dryland (75% FC) (**Figure 10A**), which is similar to the results of Mohtashami et al. (2023) on canola, where drought conditions increased the GB content. This contrasts with the findings of Ahmed et al. (2019) on wheat, where they found that GB content decreased under water-limited conditions. Lanza and Reis (2021) state that using Se in small amounts is beneficial for plants because it speeds up biological processes under both controlled and stressed conditions. As shown in **Table 4**, the results of this study also indicated that applying Se directly to the leaves of water-stressed sunflower plants increased the amount of GB. However, the increase was not very significant.

According to Sehar et al. (2021), adding Se to plants during drought stress increased the plants’ ability to adjust to changes in osmotic tension and also protected them from ROS production by directly inhibiting the formation of hydroxyl radicals and singlet oxygen. The results showed that GB could improve membrane integrity by increasing the removal of ROS and decreasing lipid peroxidation. This would protect subcellular organelles from the damage caused by drought. According to Sita et al. (2023), GB regulates antioxidant enzymes to modify H_2_O_2_ accumulation, preserving cell membrane integrity and improving nutrient accumulation. Higher levels of GB and antioxidant enzyme activities induced by external Se in water-limiting situations demonstrate an association between the antioxidant system and GB deposition. Yazdandoost Hamedani et al. (2019) demonstrated that Se supplementation improved the GB level in sunflower seedlings, which subsequently improved the water status of stressed plants. On the other hand, Mansour and Ali (2017) suggested that GB buildup is not an adaptive response, but only a sign of stress. Our current research has shown that Se-treated plants have increased GB activity, indicating a possible connection with better membrane integrity and, consequently, enhanced nutrient accumulation. Furthermore, in dryland conditions (75% FC and 50% FC), we observed decreased GB levels and amplified the activities of CAT, POD, and SOD compared with 100% FC conditions.

When a plant is stressed by drought, its metabolism responds through the production of ROS. Owing to high ROS (H_2_O_2_) generation in water-restricted conditions, these enzymes may have been activated. Semida et al. (2023) reported that Se can regulate the plant’s water content when it is hydrated, suggesting that it may defend the plant by enhancing root water absorption. The antioxidant properties of Se help plants grow and thrive, particularly under stressed conditions. Higher rates of POD and CAT activity (**Figures 9B, C** respectively) in water-stressed plants may be a way for plants to respond in adverse conditions. Rasheed et al. (2021) suggested that plants’ low POD activity might not be able to handle stress because lipid peroxidation reduces membrane permeability.

The results of Khalofah et al. (2021) in *Chenopodium quinoa* are consistent with the higher activation/levels of CAT, POD, and SOD seen in this investigation and in Kaur and Sharma (2018) in wheat. When Se was applied to cauliflower during drought stress, Hachmann et al. (2019) observed comparable outcomes and reported an increase in antioxidant activity. The appropriate amounts of Se cause O_2_^−^ to spontaneously change into H_2_O_2_ or allow Se molecules to directly quench O_2_^−^ and OH^−^. High amounts of H_2_O_2_ are reduced in plants grown under stressed conditions by activating antioxidants, especially H_2_O_2_ quenchers like GSH-Px (Lanza and Reis, 2021). By altering plant metabolism, such as antioxidant activity, Rady et al. (2020) suggested that using Se or micronutrients alone, but not together, can mitigate the harmful effects of drought stress. However, high amounts of Se in aerial spray could damage the leaf surface and reduce the activity of POD.

## 5 Conclusion

The current study focuses on how applying Se to the leaves of sunflowers can alter their morphology, function, and biochemistry during drought stress. Depending on the Se treatments, the sunflower exhibited a broad spectrum of responses. Spraying sunflowers with 30–90 ppm pure Se (Na_2_SeO_4_) under various drought stress conditions caused Se accumulation in the plants. Based on the above results, we can conclude that applying Se directly to the leaves of sunflower plants greatly increased their ability to absorb certain nutrients (Se, Na^+^, K^+^, and Ca^2+^). This was achieved by maintaining the plants’ turgidity and enhancing their physio-biochemical activities, regardless of water availability. The amount of Se in the shoots of drought-stressed plants increased from almost nothing to 93.86% when Se was applied to the leaves. In addition, it raised the production of GB and Pro by 74.46% and 68.63%, respectively. It also decreased the production of ROS by 20.89% because CAT (49.87%), POD (100.20%), and SOD (157.63%) activities were significantly improved. On the other hand, secondary metabolites such as Ant, Flv, TP, and AA levels increased during drought stress by 83.73%, 73.16%, 39.34%, and 25.51%, respectively, without any signs of Se toxicity in plants. Based on our data, our hypothesis was correct, and these results also suggest that this method could help improve the growth of Se-enriched sunflowers under water-deficient conditions. However, to determine the molecular pathways responsible for these modifications, additional research is required.

## Data availability statement

The original contributions presented in the study are included in the article/supplementary material. Further inquiries can be directed to the corresponding author.

## Author contributions

MAm: Data curation, Formal analysis, Investigation, Methodology, Writing – original draft. MZ: Methodology, Supervision, Validation, Writing – review & editing. HN: Methodology, Writing – review & editing, Visualization. MN: Methodology, Investigation, Writing – original draft. AM: Conceptualization, Supervision, Validation, Writing – review & editing. AS: Formal analysis, Software, Writing – review & editing. BK: Formal analysis, Methodology, Writing – review & editing. BA: Project administration, Software, Writing – review & editing. MAl: Project administration, Validation, Visualization, Writing – review & editing. MS: Funding acquisition, Project administration, Resources, Visualization, Writing – review & editing.

## Glossary

**Table:**

Ant	anthocyanin
AsA	ascorbic acid
Ca^2+^	calcium ions
Car	carotenoids
CAT	catalase
Cl^−^	chloride ions
Chla	chlorophyll *a*
Chlb	chlorophyll *b*
ChlR	chlorophyll ratio
df	degrees of freedom
DS	drought stress
DS0	drought stress with 100% FC
DS1	drought stress with 75% FC
DS2	drought stress with 50% FC
EC	electrical conductivity
FC	field capacity
Flv	flavonoids
GSH-Px	glutathione peroxidase
GB	glycine betaine
H_2_O_2_	hydrogen peroxide
IPCC	Intergovernmental Panel on Climate Change
Mg^2+^	magnesium
Mo	molybdenum
NO_3_^−^	nitrate
NOB	number of branches
NOL	number of leaves
NUE	nutrient use efficiency
POD	peroxidase
PS-II	photosystem-II
PBM	plant biomass
PH	plant height
K^+^	potassium ions
Pro	total soluble protein
ROS	reactive oxygen species
RFW	root fresh weight
RCa	root calcium
RDW	root dry weight
RL	root length
RK	root potassium
RNa	root sodium
rWC	root water content
Se0	0 ppm selenium
Se1	30 ppm selenium
Se2	60 ppm selenium
Se3	90 ppm selenium
Se	selenium
SE	standard error
SCa	shoot calcium
SDW	shoot dry weight
SFW	shoot fresh weight
Si	silicon
SL	shoot length
SK	shoot potassium
SSe	shoot selenium
SNa	shoot sodium
sWC	shoot water content
Na^+^	sodium ions
SOD	superoxide dismutase
TChl	total chlorophyll
TDM	total dry matter
TP	total phenolics
*p* ≤ 0.05	*significant
*p* ≤ 0.01	**significant
*p* ≤ 0.001	***significant
ns	*p* > 0.05

## References

[B1] Ahmad M. Naqve M. Lihong W. Zia M. A. Mahmood A. Javaid M. M.. (2024). Mitigating negative impact of salinity on berseem (*Trifolium alexandrinum*) by foliar application of salicylic acid. Not. Bot. Horti. Agrobo. 52, 13467. doi: 10.15835/nbha52113467

[B2] Ahmad Z. Waraich E. A. Barutcular C. Alharby H. Bamagoos A. Kizilgeci F.. (2020). Enhancing drought tolerance in *Camelina sativa* L. and canola (*Brassica napus* L.) through application of selenium. Pak. J. Bot. 52, 1927–1939. doi: 10.30848/PJB2020-6(31

[B3] Ahmed N. Zhang B. Bozdar B. Chachar S. Rai M. Li J.. (2023). The power of magnesium: unlocking the potential for increased yield, quality, and stress tolerance of horticultural crops. Front. Plant Sci. 14. doi: 10.3389/fpls.2023.1285512 PMC10628537 37941670

[B4] Ahmed N. Zhang Y. Yu H. Gabar A. Zhou Y. Li Z.. (2019). Seed priming with Glycine betaine improve seed germination characteristics and antioxidant capacity of wheat (*Triticum aestivum* L.) seedlings under water-stress conditions. Appl. Ecol. Environ. Res. 17, p8333. doi: 10.15666/aeer/1704_83338350

[B5] Alexieva V. Sergiev I. Mapelli S. Karanov E. (2001). The effect of drought and ultraviolet radiation on growth and stress markers in pea and wheat. Plant Cell Environ. 24, 1337–1344. doi: 10.1046/j.1365-3040.2001.00778.x

[B6] Al-Jaberi A. J. Abdulla A. A. Shano H. S. (2019). Effect of Seeds Soaking with Molybdenum, Spraying with Selenium and Interaction Between them on Growth and Yield of Green Peas (*Pisum sativum* L.). Basrah J. Agric. Sci. 32, 220–230. doi: 10.37077/25200860.2019.270

[B7] Ameen M. Mahmood A. Ahmad M. Mansoor Javaid M. Nadeem M. A. Asif M.. (2023a). “Impacts of climate change on fruit physiology and quality,” in Climate-resilient agriculture, vol 1: crop responses and agroecological perspectives. Ed. Hasanuzzaman M. (Springer International Publishing, Cham), 93–124.

[B8] Ameen M. Mahmood A. Sahkoor A. Zia M. A. Ullah M. S. (2024). The role of endophytes to combat abiotic stress in plants. Plant Stress 12, 100435. doi: 10.1016/j.stress.2024.100435

[B9] Ameen M. Zafar A. Javaid M. M. Zia M. A. Mahmood A. Naqve M.. (2023b). “Climate-resilient technology for maize production,” in Climate-resilient agriculture, vol 2: agro-biotechnological advancement for crop production. Ed. Hasanuzzaman M. (Springer International Publishing, Cham), 157–188.

[B10] Arikan B. Ozfidan-Konakci C. Alp F. N. Zengin G. Yildiztugay E. (2022). Rosmarinic acid and hesperidin regulate gas exchange, chlorophyll fluorescence, antioxidant system and the fatty acid biosynthesis-related gene expression in *Arabidopsis thaliana* under heat stress. Phytochem. 198, 113157. doi: 10.1016/j.phytochem.2022.113157 35271935

[B11] Arnon D. I. (1949). Copper enzymes in isolated chloroplasts. Polyphenoloxidase in *beta vulgaris* . Plant Physiol. 24, 1–15. doi: 10.1104/pp.24.1.1 16654194 PMC437905

[B12] Arshad A. Ghani M. U. Hassan M.U. Qamar H. Zubair M. (2020). “Sunflower modelling: A review,” in Systems modeling. Ed. Ahmed M. (Springer, Singapore), 307–326.

[B13] Azeem M. Sultana R. Mahmood A. Qasim M. Siddiqui Z. S. Mumtaz S.. (2023). Ascorbic and salicylic acids vitalized growth, biochemical responses, antioxidant enzymes, photosynthetic efficiency, and ionic regulation to alleviate salinity stress in *sorghum bicolor* . J. Plant Growth Regul. 42, 5266–5279. doi: 10.1007/s00344-023-10907-2

[B14] Barrs H. Weatherley P. (1962). A re-examination of the relative turgidity technique for estimating water deficits in leaves. Aust. J. Biol. Sci. 15, 413–428. doi: 10.1071/BI9620413

[B15] Bhatt T. Patel K. (2020). Carotenoids: potent to prevent diseases review. Nat. Prod. Bioprospect. 10, 109–117. doi: 10.1007/s13659-020-00244-2 32405969 PMC7253555

[B16] Bocchini M. D’Amato R. Ciancaleoni S. Fontanella M. C. Palmerini C. A. Beone G. M.. (2018). Soil selenium (Se) biofortification changes the physiological, biochemical and epigenetic responses to water stress in *zea mays* L. by inducing a higher drought tolerance. Front. Plant Sci. 9. doi: 10.3389/fpls.2018.00389 PMC5880925 29636765

[B17] Bradford M. M. (1976). A rapid and sensitive method for the quantitation of microgram quantities of protein utilizing the principle of protein-dye binding. Anal. Biochem. 72, 248–254. doi: 10.1016/0003-2697(76)90527-3 942051

[B18] Bremner J. M. (1960). Determination of nitrogen in soil by the Kjeldahl method. J. Agric. Sci. 55, 11–33. doi: 10.1017/S0021859600021572

[B19] Chance B. Maehly A. C. (1955). “Assay of catalases and peroxidases,” in Methods in enzymology (New York: Academic Press), 764–775. doi: 10.1016/S0076-6879(55)02300-8

[B20] Chapman H. D. Pratt P. F. (1962). Methods of analysis for soils, plants and waters. Soil Sci. 93, 68. doi: 10.1097/00010694-196201000-00015

[B21] Chauhan R. Awasthi S. Tripathi P. Mishra S. Dwivedi S. Niranjan A.. (2017). Selenite modulates the level of phenolics and nutrient element to alleviate the toxicity of arsenite in rice (*Oryza sativa* L.). Ecotoxicol. Environ. Saf. 138, 47–55. doi: 10.1016/j.ecoenv.2016.11.015 28006731

[B22] Davis K. (1976). Social responsibility is inevitable. Calif. Manage. Rev. 19, 14–20. doi: 10.2307/41164678

[B23] Dhami N. Cazzonelli C. I. (2020). Environmental impacts on carotenoid metabolism in leaves. Plant Growth Regul. 92, 455–477. doi: 10.1007/s10725-020-00661-w

[B24] Dietz K.-J. Zörb C. Geilfus C.-M. (2021). Drought and crop yield. Plant Biol. 23, 881–893. doi: 10.1111/plb.13304 34396653

[B25] Fan S. Wu H. Gong H. Guo J. (2022). The salicylic acid mediates selenium-induced tolerance to drought stress in tomato plants. Sci. Hortic. 300, 111092. doi: 10.1016/j.scienta.2022.111092

[B26] Franco-Navarro J. D. Brumós J. Rosales M. A. Cubero-Font P. Talón M. Colmenero-Flores J. M. (2016). Chloride regulates leaf cell size and water relations in tobacco plants. J. Exp. Bot. 67, 873–891. doi: 10.1093/jxb/erv502 26602947 PMC4737079

[B27] Franco-Navarro J. D. Díaz-Rueda P. Rivero-Núñez C. M. Brumós J. Rubio-Casal A. E. de Cires A.. (2021). Chloride nutrition improves drought resistance by enhancing water deficit avoidance and tolerance mechanisms. J. Exp. Bot. 72, 5246–5261. doi: 10.1093/jxb/erab143 33783493 PMC8272566

[B28] Ghorai M. Kumar V. Kumar V. Al-Tawaha A. R. Shekhawat M. S. Pandey D. K.. (2022). Beneficial role of selenium (Se) biofortification in developing resilience against potentially toxic metal and metalloid stress in crops: recent trends in genetic engineering and omics approaches. J. Soil Sci. Plant Nutr. 22, 2347–2377. doi: 10.1007/s42729-022-00814-y

[B29] Ghouri F. Ali Z. Naeem M. Ul-Allah S. Babar M. Baloch F. S.. (2022). Effects of silicon and selenium in alleviation of drought stress in rice. Silicon 14, 5453–5461. doi: 10.1007/s12633-021-01277-z

[B30] Giannopolitis C. N. Ries S. K. (1977). Superoxide dismutases: I. Occurrence in higher plants 1 2. Plant Physiol. 59, 309–314. doi: 10.1104/pp.59.2.309 16659839 PMC542387

[B31] Grieve C. M. Grattan S. R. (1983). Rapid assay for determination of water soluble quaternary ammonium compounds. Plant Soil 70, 303–307. doi: 10.1007/BF02374789

[B32] Groth S. Budke C. Neugart S. Ackermann S. Kappenstein F.-S. Daum D.. (2020). Influence of a selenium biofortification on antioxidant properties and phenolic compounds of apples (*Malus domestica*). Antioxidants 9, 187. doi: 10.3390/antiox9020187 32102431 PMC7070929

[B33] Gull T. Mahmood A. Shaheen C. Javaid M. M. Zia M. A. Naqve M.. (2023). “Climate change and nutrient use efficiency of plants,” in Climate-resilient agriculture: crop responses and agroecological perspectives. Ed. Hasanuzzaman M. (Springer International Publishing, Cham), 291–312.

[B34] Habibi G. (2017). Physiological, photochemical and ionic responses of sunflower seedlings to exogenous selenium supply under salt stress. Acta Physiol. Plant 39, 213. doi: 10.1007/s11738-017-2517-3

[B35] Hachmann T. L. Rezende R. Matumoto-Pintro P. T. Saath R. Anjo F. A. Menezes C. S. L. (2019). Yield, antioxidant activity and shelf-life of cauliflower inflorescences under drought stress and foliar spraying of selenium. Ciênc. Agrotec. 43, e017819. doi: 10.1590/1413-7054201943017819

[B36] Haghighi M. Sheibanirad A. Pessarakli M. (2016). Effects of selenium as a beneficial element on growth and photosynthetic attributes of greenhouse cucumber. J. Plant Nutr. 39, 1493–1498. doi: 10.1080/01904167.2015.1109116

[B37] Han D. Tu S. Dai Z. Huang W. Jia W. Xu Z.. (2022). Comparison of selenite and selenate in alleviation of drought stress in *Nicotiana tabacum* L. Chemosphere 287, 132136. doi: 10.1016/j.chemosphere.2021.132136 34492417

[B38] Hasanuzzaman M. Bhuyan M. H. M. B. Zulfiqar F. Raza A. Mohsin S. M. Mahmud J. A.. (2020). Reactive oxygen species and antioxidant defense in plants under abiotic stress: revisiting the crucial role of a universal defense regulator. Antioxidants 9, 681. doi: 10.3390/antiox9080681 32751256 PMC7465626

[B39] Hawrylak-Nowak B. Matraszek R. Pogorzelec M. (2015). The dual effects of two inorganic selenium forms on the growth, selected physiological parameters and macronutrients accumulation in cucumber plants. Acta Physiol. Plant 37, 41. doi: 10.1007/s11738-015-1788-9

[B40] Hossain A. Skalicky M. Brestic M. Maitra S. Sarkar S. Ahmad Z.. (2021). Selenium biofortification: roles, mechanisms, responses and prospects. Molecules 26, 881. doi: 10.3390/molecules26040881 33562416 PMC7914768

[B41] Huang C. Yu M. Sun L. Qin N. Wei L. (2020). Physiological responses of sweet potato seedlings under drought-stress conditions with selenium applications. J. Agric. Crop Res. 8, 98–112. doi: 10.33495/jacr_v8i5.20.129

[B42] IPCC (2012). Managing the risks of extreme events and disasters to advance climate change adaptation: special report of the intergovernmental panel on climate change (Cambridge: Cambridge University Press).

[B43] IPCC (2014). Climate change 2014 – impacts, adaptation and vulnerability: global and sectoral aspects (Cambridge: Cambridge University Press).

[B44] IPCC (2022a). Climate change and land: IPCC special report on climate change, desertification, land degradation, sustainable land management, food security, and greenhouse gas fluxes in terrestrial ecosystems (Cambridge: Cambridge University Press).

[B45] IPCC (2022b). “Contributors to the IPCC special report on global warming of 1.5°C,” in *global warming of 1.5°C* ,” in IPCC special report on impacts of global warming of 1.5°C above pre-industrial levels in context of strengthening response to climate change, sustainable development, and efforts to eradicate poverty (Cambridge University Press, Cambridge), 573–580. C. Intergovernmental Panel on Climate.

[B46] Ivanov S. Austin J. Berg R. H. Harrison M. J. (2019). Extensive membrane systems at the host–arbuscular mycorrhizal fungus interface. Nat. Plants 5, 194–203. doi: 10.1038/s41477-019-0364-5 30737512

[B47] Jiang C. Zu C. Lu D. Zheng Q. Shen J. Wang H.. (2017). Effect of exogenous selenium supply on photosynthesis, Na+ accumulation and antioxidative capacity of maize (*Zea mays* L.) under salinity stress. Sci. Rep. 7, 42039. doi: 10.1038/srep42039 28169318 PMC5294586

[B48] Kalyane D. Choudhary D. Polaka S. Goykar H. Karanwad T. Rajpoot K.. (2022). Reactive oxygen nano-generators for cancer therapy. Prog. Mater. Sci. 130, 100974. doi: 10.1016/j.pmatsci.2022.100974

[B49] Kamran M. Parveen A. Ahmar S. Malik Z. Hussain S. Chattha M. S.. (2020). An overview of hazardous impacts of soil salinity in crops, tolerance mechanisms, and amelioration through selenium supplementation. Int. J. Mol. Sci. 21, 148. doi: 10.3390/ijms21010148 PMC6981449 31878296

[B50] Kaur M. Sharma S. (2018). Influence of selenite and selenate on growth, leaf physiology and antioxidant defense system in wheat (*Triticum aestivum* L.). J. Sci. Food Agric. 98, 5700–5710. doi: 10.1002/jsfa.9117 29736998

[B51] Khalofah A. Migdadi H. El-Harty E. (2021). Antioxidant enzymatic activities and growth response of quinoa (*Chenopodium quinoa* willd) to exogenous selenium application. Plants 10, 719. doi: 10.3390/plants10040719 33917228 PMC8068041

[B52] Khorasaninejad S. Mousavi A. Soltanloo H. Hemmati K. Khalighi A. (2011). The effect of drought stress on growth parameters, essential oil yield and constituent of Peppermint (*Mentha piperita* L.). J. Med. Plant Res. 5, 5360–5365.

[B53] Lanza M. G. D. B. Reis A. (2021). Roles of selenium in mineral plant nutrition: ROS scavenging responses against abiotic stresses. Plant Physiol. Biochem. 164, 27–43. doi: 10.1016/j.plaphy.2021.04.026 33962229

[B54] Lei B. Bian Z.-h. Yang Q.-c. Wang J. Cheng R.-f. Li K.. (2018). The positive function of selenium supplementation on reducing nitrate accumulation in hydroponic lettuce (*Lactuca sativa* L.). J. Integr. Agric. 17, 837–846. doi: 10.1016/S2095-3119(17)61759-3

[B55] Li P. Zhu Y. Song X. Song F. (2020). Negative effects of long-term moderate salinity and short-term drought stress on the photosynthetic performance of Hybrid Pennisetum. Plant Physiol. Biochem. 155, 93–104. doi: 10.1016/j.plaphy.2020.06.033 32745934

[B56] Liu H. Xiao C. Qiu T. Deng J. Cheng H. Cong X.. (2023). Selenium regulates antioxidant, photosynthesis, and cell permeability in plants under various abiotic stresses: A review. Plants 12, 44. doi: 10.3390/plants12010044 PMC9824017 36616173

[B57] Lopez-Cantu D. O. González-González R. B. Sharma A. Bilal M. Parra-Saldívar R. Iqbal H. M. N. (2022). Bioactive material-based nanozymes with multifunctional attributes for biomedicine: Expanding antioxidant therapeutics for neuroprotection, cancer, and anti-inflammatory pathologies. Coord. Chem. Rev. 469, 214685. doi: 10.1016/j.ccr.2022.214685

[B58] Lu Y. Zeng F. J. Li X. Y. Zhang B. (2021). Physiological changes of three woody plants exposed to progressive salt stress. Photosynthetica 59, 171–184. doi: 10.32615/ps.2021.007

[B59] Luo H. Xing P. Liu J. Pan S. Tang X. Duan M. (2021). Selenium improved antioxidant response and photosynthesis in fragrant rice (*Oryza sativa* L.) seedlings during drought stress. Physiol. Mol. Biol. Plants 27, 2849–2858. doi: 10.1007/s12298-021-01117-9 35035140 PMC8720130

[B60] Mancinelli A. L. (1984). Photoregulation of anthocyanin synthesis. Plant Physiol. 75 (2), 447–453. doi: 10.1104/pp.75.2.447. 1: VIII. PMC1066927 16663641

[B61] Mansour M. M. F. Ali E. F. (2017). Glycinebetaine in saline conditions: an assessment of the current state of knowledge. Acta Physiol. Plant 39, 56. doi: 10.1007/s11738-017-2357-1

[B62] Mathobo R. Marais D. Steyn J. M. (2017). The effect of drought stress on yield, leaf gaseous exchange and chlorophyll fluorescence of dry beans (*Phaseolus vulgaris* L.). Agric. Water Manage. 180, 118–125. doi: 10.1016/j.agwat.2016.11.005

[B63] Mehlich A. (1953). “Determination of P, ca, mg, K, na and NH4,” in Short test methods used in soi testing division (Department of Agriculture, Raleigh, North Caroline), 23–89.

[B64] Michaletti A. Naghavi M. R. Toorchi M. Zolla L. Rinalducci S. (2018). Metabolomics and proteomics reveal drought-stress responses of leaf tissues from spring-wheat. Sci. Rep. 8, 5710. doi: 10.1038/s41598-018-24012-y 29632386 PMC5890255

[B65] Mohammed H. A. (2018). Effect of Exogenous Application of Zinc and Selenium on Quality Characteristic for Sunflower plant under water stress. Plant Arch. 18, 2661–2671.

[B66] Mohtashami R. Movahhedi Dehnavi M. Balouchi H. Farajee H. (2023). Improving physiological and biochemical responses of dryland canola by selenium foliar application and supplemental irrigation. Acta Physiol. Plant 45, 47. doi: 10.1007/s11738-023-03532-9

[B67] Mounicou S. Vonderheide A. P. Shann J. R. Caruso J. A. (2006). Comparing a selenium accumulator plant (Brassica juncea) to a nonaccumulator plant (*Helianthus annuus*) to investigate selenium-containing proteins. Anal. Bioanal. Chem. 386, 1367–1378. doi: 10.1007/s00216-006-0707-8 16933129

[B68] Mroczek-Zdyrska M. Strubińska J. Hanaka A. (2017). Selenium Improves Physiological Parameters and Alleviates Oxidative Stress in Shoots of Lead-Exposed *Vicia faba* L. minor Plants Grown Under Phosphorus-Deficient Conditions. J. Plant Growth Regul. 36, 186–199. doi: 10.1007/s00344-016-9629-7

[B69] Mukherjee S. P. Choudhuri M. A. (1983). Implications of water stress-induced changes in the levels of endogenous ascorbic acid and hydrogen peroxide in Vigna seedlings. Physiol. Plant 58, 166–170. doi: 10.1111/j.1399-3054.1983.tb04162.x

[B70] Mukhtiar A. Lihong W. Mahmood A. Ameen M. Zia M. A. Arshad T.. (2023). The role of endophytes and rhizobacteria to combat drought stress in wheat. Not. Bot. Horti. Agrobo. 51, 13453. doi: 10.15835/nbha51413453

[B71] Munns R. Passioura J. B. Colmer T. D. Byrt C. S. (2020). Osmotic adjustment and energy limitations to plant growth in saline soil. New Phytol. 225, 1091–1096. doi: 10.1111/nph.15862 31006123

[B72] Nadler A. Frenkel H. (1980). Determination of soil solution electrical conductivity from bulk soil electrical conductivity measurements by the four-electrode method. Soil Sci. Soc Am. J. 44, 1216–1221. doi: 10.2136/sssaj1980.03615995004400060017x

[B73] Naseem M. B. B. ALI Q. Ali S. Khalid M. R. Nawaz M. (2023). Selenium application reduces cadmium uptake in tomato (*Lycopersicum esculentum* Mill.) by modulating growth, nutrient uptake, gas exchange, root exudates and antioxidant profile. Pak. J. Bot. 55, 1633–1646. doi: 10.30848/PJB2023-5(6

[B74] Nawaz F. Ahmad R. Ashraf M. Y. Waraich E. A. Khan S. Z. (2015). Effect of selenium foliar spray on physiological and biochemical processes and chemical constituents of wheat under drought stress. Ecotoxicol. Environ. Saf. 113, 191–200. doi: 10.1016/j.ecoenv.2014.12.003 25499052

[B75] Nazim M. Ali M. Li X. Anjum S. Ahmad F. Zulfiqar U.. (2023). Unraveling the synergistic effects of microbes and selenium in alleviating drought stress in *Camelina sativa* L. Plant Stress 9, 100193. doi: 10.1016/j.stress.2023.100193

[B76] Nelson D. W. Sommers L. E. (1982). “Total carbon, organic carbon, and organic matter,” in Methods of soil analysis (Wiley Online Library, USA), 539–579.

[B77] Pedron F. N. Messias A. Zeida A. Roitberg A. E. Estrin D. A. (2023). Novel lennard-jones parameters for cysteine and selenocysteine in the AMBER force field. J. Chem. Inf. Model. 63, 595–604. doi: 10.1021/acs.jcim.2c01104 36630702

[B78] Rady M. M. Belal H. E. E. Gadallah F. M. Semida W. M. (2020). Selenium application in two methods promotes drought tolerance in Solanum lycopersicum plant by inducing the antioxidant defense system. Sci. Hortic. 266, 109290. doi: 10.1016/j.scienta.2020.109290

[B79] Rasheed F. Gondal A. Kudus K. A. Zafar Z. Nawaz M. F. Khan W. R.. (2021). Effects of soil water deficit on three tree species of the arid environment: variations in growth, physiology, and antioxidant enzyme activities. Sustainability 13, 3336. doi: 10.3390/su13063336

[B80] Rasheed A. Li H. Tahir M. M. Mahmood A. Nawaz M. Shah A. N.. (2022). The role of nanoparticles in plant biochemical, physiological, and molecular responses under drought stress: A review. Front. Plant Sci. 13. doi: 10.3389/fpls.2022.976179 PMC9730289 36507430

[B81] Rasool A. Hafiz Shah W. Padder S. A. Tahir I. Alharby H. F. Hakeem K. R.. (2023). Exogenous selenium treatment alleviates salinity stress in Proso Millet (*Panicum miliaceum* L.) by enhancing the antioxidant defence system and regulation of ionic channels. Plant Growth Regul. 100, 479–494. doi: 10.1007/s10725-022-00826-9

[B82] Revanasiddappa H. Kiran Kumar T. (2002). Spectrophotometric determination of selenium by use of thionin. Anal. Bioanal. Chem. 374, 1121–1124. doi: 10.1007/s00216-002-1581-7 12458430

[B83] Rolnik A. Olas B. (2021). The plants of the asteraceae family as agents in the protection of human health. Int. J. Mol. Sci. 22, 3009. doi: 10.3390/ijms22063009 33809449 PMC7999649

[B84] Rosales M. A. Franco-Navarro J. D. Peinado-Torrubia P. Díaz-Rueda P. Álvarez R. Colmenero-Flores J. M. (2020). Chloride improves nitrate utilization and NUE in plants. Front. Plant Sci. 11. doi: 10.3389/fpls.2020.00442 PMC7264407 32528483

[B85] Sardari M. Rezayian M. Niknam V. (2022). Comparative study for the effect of selenium and nano-selenium on wheat plants grown under drought stress. Russ. J. Plant Physl. 69, 127. doi: 10.1134/S102144372206022X

[B86] Sarkar J. Mridha D. Davoodbasha M. A. Banerjee J. Chanda S. Ray K.. (2023). A state-of-the-art systemic review on selenium nanoparticles: mechanisms and factors influencing biogenesis and its potential applications. Biol. Trace Elem. Res. 201, 5000–5036. doi: 10.1007/s12011-022-03549-0 36633786

[B87] Sehar S. Adil M. F. Zeeshan M. Holford P. Cao F. Wu F.. (2021). Mechanistic insights into potassium-conferred drought stress tolerance in cultivated and tibetan wild barley: differential osmoregulation, nutrient retention, secondary metabolism and antioxidative defense capacity. Int. J. Mol. Sci. 22, 13100. doi: 10.3390/ijms222313100 34884904 PMC8658718

[B88] Semida W. M. Abd El-Mageed T. A. Gyushi M. A. H. Abd El-Mageed S. A. Rady M. M. Abdelkhalik A.. (2023). Exogenous selenium improves physio-biochemical and performance of drought-stressed phaseolus vulgaris seeded in saline soil. Soil Syst. 7, 67. doi: 10.3390/soilsystems7030067

[B89] Shalaby T. Bayoumi Y. Alshaal T. Elhawat N. Sztrik A. El-Ramady H. (2017). Selenium fortification induces growth, antioxidant activity, yield and nutritional quality of lettuce in salt-affected soil using foliar and soil applications. Plant Soil 421, 245–258. doi: 10.1007/s11104-017-3458-8

[B90] Sharma K. Gupta S. Thokchom S. D. Jangir P. Kapoor R. (2021). Arbuscular mycorrhiza-mediated regulation of polyamines and aquaporins during abiotic stress: deep insights on the recondite players. Front. Plant Sci. 12. doi: 10.3389/fpls.2021.642101 PMC8247573 34220878

[B91] Shirazi M. A. Boersma L. (1984). A unifying quantitative analysis of soil texture. Soil Sci. Soc Am. J. 48, 142–147. doi: 10.2136/sssaj1984.03615995004800010026x

[B92] Singhal R. K. Fahad S. Kumar P. Choyal P. Javed T. Jinger D.. (2023). Beneficial elements: New Players in improving nutrient use efficiency and abiotic stress tolerance. Plant Growth Regul. 100, 237–265. doi: 10.1007/s10725-022-00843-8

[B93] Singleton V. L. Joseph A. Rossi (1965). Colorimetry of total phenolics with phosphomolybdic-phosphotungstic acid reagents. Am. J. Enol. Vitic. 16, 144. doi: 10.5344/ajev.1965.16.3.144

[B94] Sita K. Sehgal A. Bhardwaj A. Bhandari K. Jha U. Vara Prasad P. V.. (2023). Selenium supplementation to lentil (*Lens culinaris* Medik.) under combined heat and drought stress improves photosynthetic ability, antioxidant systems, reproductive function and yield traits. Plant Soil 486, 7–23. doi: 10.1007/s11104-022-05310-x

[B95] Snedecor G. W. (1956). Statistical methods: applied to experiments in agriculture and biology (Ames: The Iowa state college press).

[B96] Song J. Xin L. Gao F. Liu H. Wang X. (2024). Effects of foliar selenium application on oxidative damage and photosynthetic properties of greenhouse tomato under drought stress. Plants 13, 302. doi: 10.3390/plants13020302 38276758 PMC10819105

[B97] Uddin S. M. N. Rahaman M. Z. Thammi T. J. Islam M. R. Masud M. I. U. Uddin M. G.. (2022). Low serum concentration of zinc, selenium, calcium, potassium and high serum concentration of iron, sodium are associated with myocardial infarction. Aging Health Res. 2, 100063. doi: 10.1016/j.ahr.2022.100063

[B98] Wang M. Wang Y. Ge C. Wu H. Jing F. Wu S.. (2023b). Foliar selenium nanoparticles application promotes the growth of maize (*Zea mays* L.) seedlings by regulating carbon, nitrogen and oxidative stress metabolism. Sci. Hortic. 311, 111816. doi: 10.1016/j.scienta.2022.111816

[B99] Wang F. Yang J. Hua Y. Wang K. Guo Y. Lu Y.. (2023a). Transcriptome and metabolome analysis of selenium treated alfalfa reveals influence on phenylpropanoid biosynthesis to enhance growth. Plants 12, 2038. doi: 10.3390/plants12102038 37653955 PMC10224443

[B100] Wolf B. (1982). A comprehensive system of leaf analyses and its use for diagnosing crop nutrient status. Commun. Soil Sci. Plant Anal. 13, 1035–1059. doi: 10.1080/00103628209367332

[B101] Wu G.-Q. Wang C.-M. Su Y.-Y. Zhang J.-J. Feng R.-J. Liang N. (2014). Assessment of drought tolerance in seedlings of sugar beet (*Beta vulgaris* L.) cultivars using inorganic and organic solutes accumulation criteria. Soil Sci. Plant Nutr. 60, 565–576. doi: 10.1080/00380768.2014.921579

[B102] Yadav S. Sharma S. Sharma K. D. Dhansu P. Devi S. Preet K.. (2023). Selenium mediated alterations in physiology of wheat under different soil moisture levels. Sustainability 15, 1771. doi: 10.3390/su15031771

[B103] Yazdandoost Hamedani M. Ghobadi M. Ghobadi M. E. Jalali Honarmand S. Saeedi M. (2019). Influence of Foliar Application of Some Chemicals on Gas Exchange, Water Relations and Photosynthetic Traits in Sunflower (*Helianthus annuus* L.) under Different Irrigation Regimes. Iran. J. Field Crops Res. 17, 477–489. doi: 10.22067/gsc.v17i3.76843

[B104] Yin H. Qi Z. Li M. Ahammed G. J. Chu X. Zhou J. (2019). Selenium forms and methods of application differentially modulate plant growth, photosynthesis, stress tolerance, selenium content and speciation in *Oryza sativa* L. Ecotoxicol. Environ. Saf. 169, 911–917. doi: 10.1016/j.ecoenv.2018.11.080 30597791

[B105] Zafar S. Afzal H. Ijaz A. Mahmood A. Ayub A. Nayab A.. (2023). Cotton and drought stress: An updated overview for improving stress tolerance. S. Afr. J. Bot. 161, 258–268. doi: 10.1016/j.sajb.2023.08.029

[B106] Zahedi S. M. Hosseini M. S. Daneshvar Hakimi Meybodi N. Peijnenburg W. (2021). Mitigation of the effect of drought on growth and yield of pomegranates by foliar spraying of different sizes of selenium nanoparticles. J. Sci. Food Agric. 101, 5202–5213. doi: 10.1002/jsfa.11167 33608893

[B107] Zahedi S. M. Hosseini M. S. Fahadi Hoveizeh N. Kadkhodaei S. Vaculík M. (2023). Comparative morphological, physiological and molecular analyses of drought-stressed strawberry plants affected by SiO2 and SiO2-NPs foliar spray. Sci. Hortic. 309, 111686. doi: 10.1016/j.scienta.2022.111686

[B108] Zembala M. Filek M. Walas S. Mrowiec H. Kornaś A. Miszalski Z.. (2010). Effect of selenium on macro- and microelement distribution and physiological parameters of rape and wheat seedlings exposed to cadmium stress. Plant Soil 329, 457–468. doi: 10.1007/s11104-009-0171-2

[B109] Zhang L. Chu C. (2022). Selenium uptake, transport, metabolism, reutilization, and biofortification in rice. Rice 15, 30. doi: 10.1186/s12284-022-00572-6 35701545 PMC9198118

[B110] Zhang H. Zhao Z. Zhang X. Zhang W. Huang L. Zhang Z.. (2019). Effects of foliar application of selenate and selenite at different growth stages on Selenium accumulation and speciation in potato (*Solanum tuberosum* L.). Food Chem. 286, 550–556. doi: 10.1016/j.foodchem.2019.01.185 30827646

[B111] Zhishen J. Mengcheng T. Jianming W. (1999). The determination of flavonoid contents in mulberry and their scavenging effects on superoxide radicals. Food Chem. 64, 555–559. doi: 10.1016/S0308-8146(98)00102-2

[B112] Zia R. Nawaz M. S. Siddique M. J. Hakim S. Imran A. (2021). Plant survival under drought stress: Implications, adaptive responses, and integrated rhizosphere management strategy for stress mitigation. Microbiol. Res. 242, 126626. doi: 10.1016/j.micres.2020.126626 33189069

